# Investigating the Stability of Individual Carboxylate-Rich Alicyclic Molecules Under Simulated Environmental Irradiation and Microbial Incubation Conditions

**DOI:** 10.1021/acs.est.5c01958

**Published:** 2025-08-14

**Authors:** Alexander J. Craig, Mahsa Norouzi, Paul Löffler, Foon Yin Lai, Rim Mtibaà, Eva Breyer, Federico Baltar, Lindon W. K. Moodie, Jeffrey A. Hawkes

**Affiliations:** 1 Analytical Chemistry, Department of Chemistry BMC, 8097 Uppsala University, Uppsala 752 37, Sweden; 2 Drug Design and Discovery, Department of Medicinal Chemistry, 8097 Uppsala University, Uppsala 752 37, Sweden; 3 Department of Aquatic Sciences and Assessment, 8095 Swedish University of Agricultural Sciences, Box 7050, Uppsala 750 07, Sweden; 4 Fungal & Biogeochemical Oceanography Group, Department of Functional and Evolutionary Ecology, 27258 University of Vienna, Vienna 1030, Austria; 5 Fungal & Biogeochemical Oceanography Group, College of Oceanography and Ecological Science, Shanghai Ocean University, Shanghai 201306, China

**Keywords:** dissolved organic matter, carboxylate-rich alicyclic molecules, simulated irradiation, microbial incubations, stability, mass spectrometry

## Abstract

Understanding dissolved organic matter (DOM) relies on the development of methods capable of navigating its complexity. Although analytical techniques have continually advanced, the fate of individual compound classes remains nearly impossible to track with the current technology. Previously, we reported the synthesis of carboxylate-rich alicyclic molecule (CRAM) compounds that shared more similar analytical features with DOM than previously available standards. Here, we adopt an alternative approach to the conventional use of DOM as a bulk material by subjecting our synthesized CRAM compounds to simulated solar irradiation and microbial incubation experiments alongside molecules with chosen biological or chemical relevance. Irradiation experiments typically showed that compounds bearing only carboxylic acids and/or alcohols on a saturated carbon backbone were the most resistant to photochemical degradation but also that some of the investigated CRAM analogues were notably more stable in the presence of DOM. Within microbial incubations, all of our synthesized CRAMs were entirely stable after 8 months in various aquatic settings. These sets of experiments provide support for the proposed stability of CRAM within the environment as well as providing a platform from which a more diverse set of molecules can be used to assist in probing the stability of DOM.

## Graphical abstract

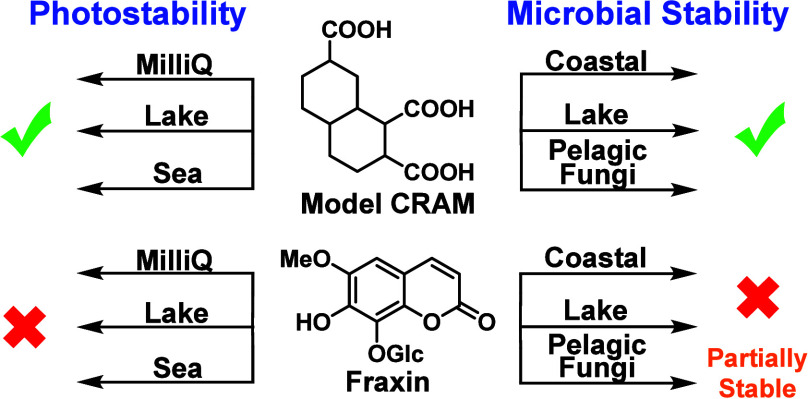

## 1 Introduction

Within all of Earth’s bodies of water, dissolved organic matter (DOM) amounts to approximately 700 gigatons of carbon.^1^ The most important role of DOM within the environment is as a vector for nutrient transport, specifically between microbes and the decomposition products of life. While the vast majority of DOM-turnover occurs through these labile DOM (LDOM) pool metabolites, any readily available materials are quickly harnessed and reused by microbes. Conversely, approximately 95% of DOM at any one point in time is designated as recalcitrant (RDOM) and has a residence half-life in the ocean of between 4000 and 6000 years.^1^ Both the sheer size and overwhelming chemical complexity of this low-reactivity pool of material has puzzled scientists for decades.^2^

The extremely long lifetime of RDOM is typically explained by three concepts.^2,3^ Intrinsic chemical stability hypothesizes that the chemical structures that DOM contains are stable to degradation and sequestration.^4^ The dilution hypothesis suggests that DOM is composed of an extremely diverse mixture of molecules present at tiny concentrations that are not viable for biological utilization, but would be if present at higher concentrations.^5^ Finally, restricted or absent essential nutrients can limit organisms from metabolizing DOM.^6,7^ Likely, it is all of these factors that contribute to the total stability of the RDOM, which is chemically diverse. Previous attempts to understand the fluxes of RDOM have investigated the photochemical,^8–12^ biological,^12–15^ and sequestrative^16–19^ processes that affect it. However, most of these types of studies have examined DOM as a bulk material, often using only chemical formula assignments to differentiate between chemical class and, by extension, molecular structure.^20–26^ This simplification can be seen as necessary due to the limitations of analytical methods and instruments but overlooks structural isomerism and its effects on stability, ultimately generalizing the findings of individual studies.

While the extensive use of mass spectrometry (MS) and nuclear magnetic resonance spectroscopy (NMR) in recent decades has improved the understanding of the chemical composition of DOM, these techniques have crucial limitations. Most importantly, both techniques provide an aggregate view of any individual DOM data set, with outputs representing averages of its total composition. NMR data is frequently bucketed into a few broad and poorly defined regions, hindering nuanced structural insight.^27^ Within MS analysis, regions in van Krevelen diagrams are frequently ascribed to classes such as sugars, proteins, or carboxylate-rich alicyclic molecules (CRAM). However, one cannot definitively assign a specific molecular formula to a chemical class based only on MS data, despite this being common in the field. Critically, isomeric compounds can exhibit radically different reactivity, even if they belong to the same chemical class, and as a result, any generalization hinders accurate assessment of the properties of DOM. Compounding this, standards of common classes of RDOM molecules are mostly unavailable by synthesis or isolation. Without accurate structural description, the understanding of the chemical behavior of individual RDOM compounds is limited to theory alone and pushes discussions on the fluxes and nature of DOM away from evidence and toward speculation.

Recently, our group disclosed the synthesis of simple CRAM analogues.^28^ As a compound class, CRAM is hypothesized as one of the largest pools of material within RDOM and consists of molecules predominantly built from fused alicyclic rings and furnished with several carboxylic acid functionalities.^29^ In our initial work, we showed that the chemical features of eight novel synthetic analogues more accurately aligned with the postulated features of the theorized environmental CRAM compound class than previously used compounds, such that they could be used in future experiments to study the recalcitrant properties of CRAM. Here, we test the stability of our first CRAM standards alongside an additional curated set of molecules with chosen biological or chemical relevance. The compounds were subjected to irradiation experiments using a solar simulator and incubations with lake and coastal water microbial communities, as well as isolated pelagic fungal strains. The behavior of the synthesized molecules alongside other pure standards in these settings provides previously inaccessible information about the stability of individual compounds with the appropriate CRAM functionality and molecular formulas.

## 2 Methods and Materials

Expanded information is available for all Methods and Materials sections in the Supporting Information (SI), including information about quality control.

### 2.1 Materials

Our eight synthetic CRAM diastereomeric mixtures were combined to comprise compounds **1**–**4** (Figure 1, purities; **1a**—92%, **1b**—99%, **2a**—90%, **2b**—98%, **3a**—99+%, **3b**—95%, **4a**—94%, and **4b**—97%,^28^ where compounds denoted **a** have *syn* stereochemistry at the 1,2-diacid, and compounds denoted **b** have *anti* stereochemistry). In addition, nine commercially available compounds were selected to represent general classes of biological molecules (Figure 1, **5**–**13**): cholic acid (**5**, 97% purity, terpenoid),^28^ oleanolic acid (**6**, >98% purity, terpenoid), Leu-Gly-Gly (**7**) (≥98%, peptide), syringic acid (**8**, ≥ 95%, phenol/tannin monomer), fraxin (**9**, ≥95%, coumarin-glycoside conjugate), glycyrrhizic acid (**10**, ≥95%, terpenoid-glycoside conjugate), raffinose (**11**, ≥98%, oligosaccharide), and guanosine monophosphate (**12,** ≥95%, nucleotide). An additional compound, 2-(4-(2,2-dicarboxy-ethyl)-2,5-dimethoxy-benzyl)-malonic acid (**13**, purity not listed), was selected as an aromatic CRAM-like equivalent. The CRAM-like compounds **1**–**4** were designed based on putative structures described by Hertkorn and colleagues,^29^ as described previously.^28^ Cholic acid (**5**), oleanolic acid (**6**), and glycyrrhizic acid (**10**) were selected as commercially available polycyclic terpenoid molecules of biological origin, which have backbone similarities to proposed CRAM scaffolds, but different functionality, including a glycoside linkage to a disaccharide moiety in the case of **10**. A peptide (**7**), sugar (**11**), and nucleotide (**12**) were included as expected labile biological metabolites, and natural products syringic acid (**8**) and fraxin (**9**) were included as aromatic plant metabolites. The aromatic carboxylate-rich molecule (**13**) was included due to its similarity to proposed CRAM chemical functionalities, and also due to its continued use as a DOM-like standard in the DOM HRMS literature.^30–34^

**Figure 1:**
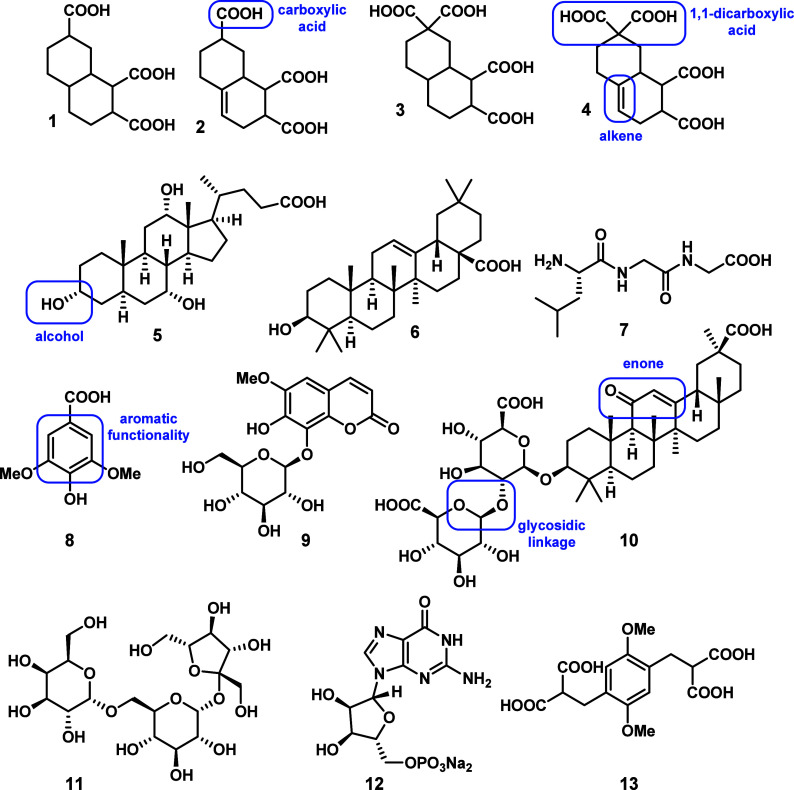
Compounds used in this study; CRAM-like diastereomeric mixtures **1**–**4** and commercially available compounds **5**–**13**. Functionality discussed later in this paper is highlighted in blue.

Four sample matrices were used in the experiments, Milli-Q water (MQ), artificial seawater (ASW),^35^ and surface lake water (LW) taken in late summer from Långsjö, near Bjorklinge, Sweden: 60°02′31.97″ N, 17°33′36.40″ E, and coastal seawater (CSW), taken in late summer from the jetty at Tjärnö Marine Laboratory, Sweden (see SI page S2). LW and CSW were filtered (0.7 μm, GF/F filter) to remove microbes.

Långsjö is regularly sampled as part of a long-term monitoring program in Sweden,^36^ and water parameters have been very stable over several years prior to our sampling. Water quality parameters were measured 9 days before our sampling date and included TOC 6.3 mg/L, total nitrogen 18 μg/L, and total phosphorus 16.4 μg/L. At Tjärnö, seawater is also measured nearby at Kosterfjörden, and in the same month as sampling, TOC was measured at 2 mg/L, nitrate+nitrite, and phosphate were measured at 2.66 and 0.08 μM (at surface, respectively).^37^

### 2.2 Preparation of Compound Mixture and Control Samples

For the 427 h solar incubation, microbial community, and isolated fungal experiments, a mixed stock solution was prepared from **1** to **13** in dimethyl sulfoxide (at 0.1 mg/mL each) initially into 50:50 methanol/water at 5 ppm concentration each, before being diluted into MQ, ASW, LW, or CSW to a concentration of 10 ppb (**5**–**13**) or 20 ppb (**1**–**4**), due to the combination of 10 ppb of each isomer (i.e., **1a** and **2a**). For the 93 h solar incubations, a mixed stock solution of **1**–**13** in MQ was diluted into MQ to a concentration of 10 ppb (**5**–**13**) or 20 ppb (**1**–**4**), avoiding organic solvents.

### 2.3 Irradiation Experiments

Irradiation experiments were conducted using a Suntest XXL+FD instrument (Atlas, Linsengericht-Altenhaßlau, Germany) at 25 °C with an irradiation intensity of 65 W m^–2^, adjusted over 300–400 nm. The chamber has three xenon lamps whose spectral distribution aligns well with the international standard CIE 85 (Figure SI1, page S3). For the first, longer experiments, samples were continuously irradiated for 427 h, compared with 93 h in the second, shorter experiment. The cumulative irradiant exposure was 10,000 and 2180 kJ m^–2^, respectively, corresponding to approximately 132 and 29 days at sea level, respectively. Additional details are included in the SI on page S2.

### 2.4 Biological Community Incubation

Samples were prepared using LW and 2% unfiltered lake water inoculum (experiments LW22 and LW259) or CSW and 2% unfiltered coastal seawater inoculum (experiment CSW251), before being placed at 20 °C in a dark, temperature-controlled room for 22 days (LW22), 259 days (LW259), and 251 days (CSW251).

### 2.5 Marine Fungi Incubation

The marine pelagic fungal cultures used were isolated from open ocean waters and included *Rhodotorula sphaerocarpa*, unknown fungal strain ECO1–30, Cladosporium sp., and *Sakaguchia dacryoidea*.^38^ Fungal liquid cultures were performed using ASW inoculated with 1% fresh fungal suspension over 102 days at 20 °C in the dark.

### 2.6 Sample Analysis

Data were exported as Thermo.raw files, and these were converted to .mzXML using ReAdW software. These data were processed with mzMine4 (batch file available in Supporting Information), and the resulting feature table was exported to a csv file.

The csv was filtered to only include the masses that corresponded to the major deprotonated and adduct peaks of the 13 compound masses spiked and one internal standard (M–H^–^ ion) to two decimal places. The correct feature was selected based on retention time, leaving 32 rows in the first analysis and 28 rows in the second analysis, because several compounds, like the synthesized CRAMs, had numerous isomers or adducts. The intensities of each compound were summed in these cases with multiple features, leaving a single row for each detected compound. The compounds not detected were raffinose, guanosine monophosphate, and oleanolic acid in all experiments, as well as syringic acid in the second set of experiments. We suspect that the former two were too hydrophilic for extraction and oleanolic acid too hydrophobic for solubility. All three also gave poor signals when analyzed at 1 ppm as standards, presumably due to the ESI settings and the compound chemistry (Figure SI4, page S7).

For the rows of data that were left after these processing steps (excluding the hippuric acid internal standard), the intensities were normalized to hippuric acid, then corrected for the original extract volume onto Agilent PPL sorbent, and then blank subtracted with the relevant blank. This was the average of three MQ water extracts for all except the lake samples, which had an unspike lake sample as the blank. The resulting values were blank subtracted and internal standard normalized “per liter” counts (eq 1). Finally, these values were normalized to the control samples in each experimental case, so that the experiment (after irradiation or incubation) averaged a value scaled to the time zero value (a percentage, eq 2). These percentages are plotted in the Section 3 along with the standard error of the difference of the calculated percent remaining (eq 3):

$$\mathrm{Compound}\;\mathrm{signal}\left(\mathrm{CS}\right)=\frac{100\times \sum_{x=1}^{x=n}i_{x}}{i_{\mathrm{hip}}\times V_{\mathrm{ex}}}$$ 1

where *i* is peak intensity, subscript *x* refers to different adducts and isomers detected, subscript hip refers to the hippuric acid internal standard, and *V*_ex_ is the extract volume. Each compound signal was averaged across three bottle replicates (for both test and control), and these sample means and standard deviations for the test and control were used to determine the % compound remaining (eq 2) and standard error of difference (eq 3), which was used as the error bar in Figures 2 and 3:

$$\mathrm{mean}\%\mathrm{remaining}=100\times \frac{\frac{\sum {\mathrm{CS}}_{\mathrm{test}}}{n_{\mathrm{test}}}}{\frac{\sum {\mathrm{CS}}_{\mathrm{control}}}{n_{\mathrm{control}}}}$$ 2

$$\%\mathrm{Standard}\;\mathrm{error}\;\mathrm{of}\;\mathrm{difference}=100\times \frac{\sqrt{\frac{\mathrm{stdev}{\left(CS_{\mathrm{test}}\right)}^{2}}{n_{\mathrm{test}}}+\frac{s\mathrm{tdev}{\left({\mathrm{CS}}_{\mathrm{control}}\right)}^{2}}{n_{\mathrm{control}}}}}{\frac{\sum {\mathrm{CS}}_{\mathrm{control}}}{n_{\mathrm{control}}}}$$ 3

Changes to HRMS data from lake water were evaluated using %Bray–Curtis dissimilarity (%BC), based on eq 4, where signal intensity *I* is compared between sample *p* and *q* for each molecular mass *k* (from *k*_1_ to *k*_n_):

$$\%\mathrm{BC}=100\frac{\sum_{k=1}^{n}\left|I_{p,k}-I_{q,k}\right|}{\sum_{k=1}^{n}\left|I_{p,k}+I_{q,k}\right|}$$ 4

Additional procedures for extraction, LCMS analysis, and data analysis for all experiments are provided in SI (page S5), with the data processing workflow (Figure SI2, page S6) and an example (Figure SI3, page S6 included) as well as calibration curves for all tested compounds against hippuric acid (Figure SI5, page S8).

### 2.7 Experimental Considerations

Of the 13 compounds subjected to testing, it was found that **6**, **11**, and **12** were not observed in any control or postexperiment samples (while still being observed prior to extraction, see Figure SI4, page S7). For **11** and **12**, this is likely due to their hydrophilicity, preventing their retention during solid-phase extraction (SPE) extraction. Conversely, **6** likely was too insoluble in water to be present in sufficient amounts for analytical detection.^39^ Additionally, **8** was not observed in either control or post-experiment samples for either the 250-day LW incubations or the 100-day fungal incubations, likely due to poor ionization efficiency. The analytical experimental values shown in Figures 2–4 have only moderate precision and accuracy due to various factors, including (1) biological variability and general bottle effects, (2) the summing of several isomers for a single reported value, (3) lack of accurate internal standards for quantification, (4) the experiments were performed on a mixture, and (5) the low concentrations used. Precision was only moderate in these experiments, with relative standard deviations between normalized triplicates averaging 27 and 37% in the shorter incubations and irradiation experiments and longer incubations, respectively. For these reasons, we focus our discussion on whether individual compounds were largely unaffected, were partially degraded, or were completely removed.

## 3 Results and Discussion

### 3.1 Irradiation Experiments

In the first experiment, compounds **1**–**13** were irradiated for 427 h under simulated solar conditions. Here, we chose the longest experiment time that was practically feasible based on equipment availability, to test for stability over the longest possible time scale. Initial stock solutions were prepared using small amounts of DMSO and methanol to attempt to solubilize hydrophobic compounds like oleanolic acid before their dilution to 10 ppb concentrations in water.

**Figure 2:**
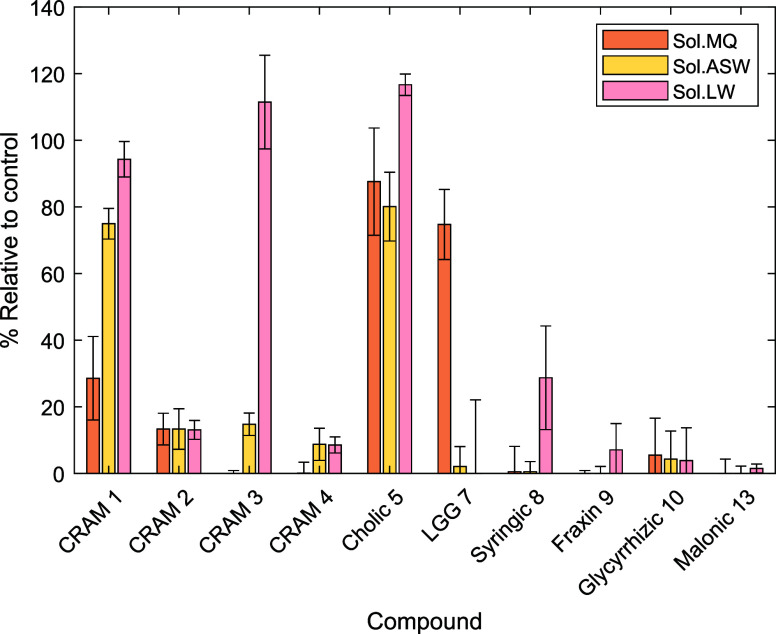
Percent remaining values for the irradiation treatments relative to time zero for 10 compound groups detected by ultrahigh performance liquid chromatography electrospray ionization high-resolution mass spectrometry (UPLC-ESI-HRMS) after solid-phase extraction. Sol.MQ, solar Milli-Q water; Sol.ASW, solar artificial seawater; Sol.LW, solar lake water. Compound names and numbers refer to those in Figure 1.

**Figure 3:**
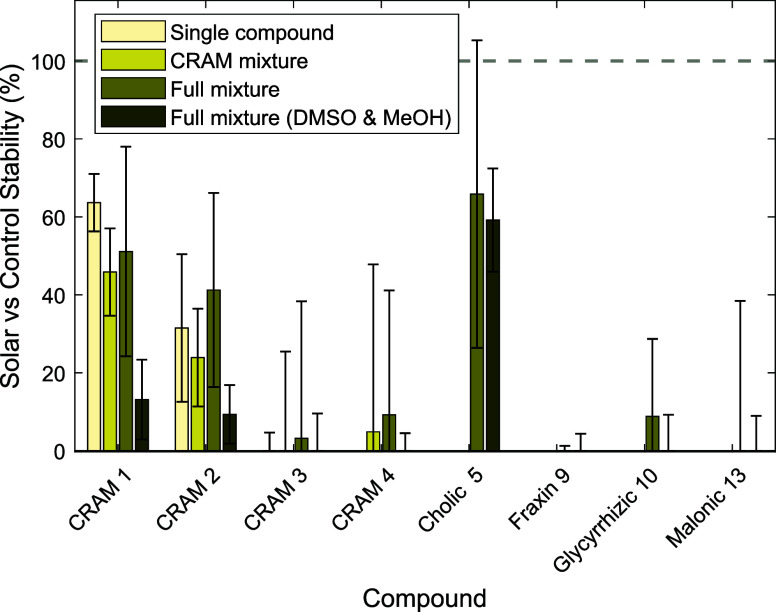
Shorter (93 h) solar experiments to check for mixture and solvent effects on CRAM compound stability. Compounds were tested individually (single compound), as a mixture of only CRAM compounds (CRAM mixture), as a mixture of all compounds **1**–**13**, and as the full mixture but with large additions of DMSO and MeOH (see main text).

**Figure 4:**
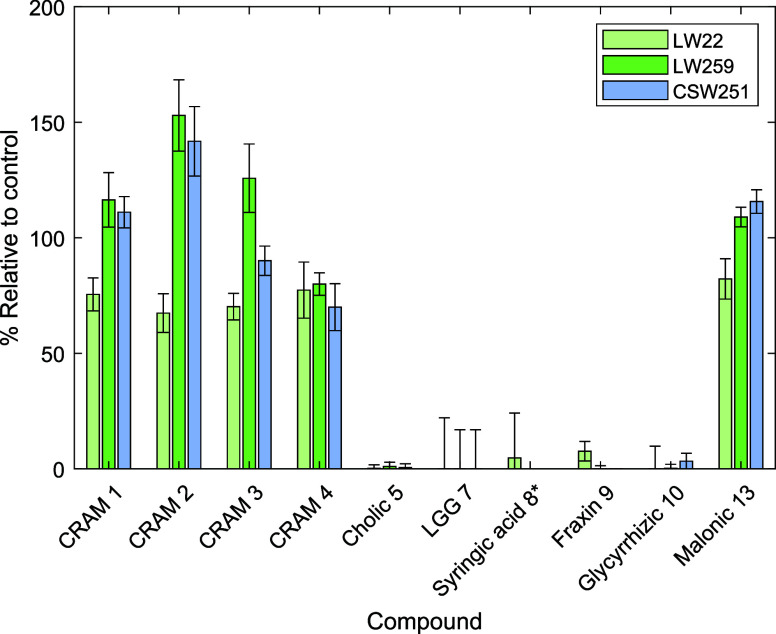
Percent remaining values compared to time zero control for 10 compound types detected by UPLC-ESI-HRMS after solid-phase extraction for the biological incubations. *Syringic acid **8** was detected only in the LW22 test and its control, and not in the LW259, CSW251, or their control samples. LW22 = lake water 22 days, LW259 = lake water 259 days, CSW251 = coastal seawater 251 days.

To interpret the results of the stability experiments, we have prepared bar graphs that show % compound remaining relative to the control samples (Figures 2 and 3 for irradiation experiments and Figure 4 for biological incubation). In these figures, the data shown was first normalized to an internal standard and then the volume of sample extracted, before averaging of intensities between three replicates and then comparing the mean value to the control mean (at time zero) as a percentage. The error bar in such a calculation is determined as the standard error of the difference of means (see Section 2.6), which is scaled to the same % scale for the graph. The error bars therefore include experimental, sample preparation, and analytical errors for both the experiment and the control samples and are larger than the standard error of either the experiment or control means, accounting for the uncertainty of comparing the two.

For the 427 h experiment in MQ (Figure 2), all compounds except for CRAMs **1** and **2**, cholic acid (**5**), and Leu-Gly-Gly (**7**) were completely degraded. For **5**, its reduced carbon backbone and seemingly unreactive carboxylic acid and alcohol functionalities appear to be stable to degradation. CRAM **1**, as the next most stable compound, contains only carboxylic acid functionalities on a fully reduced carbon backbone. As such, the greater degradation of **1** compared to **5** could be attributed to either its increased number of carboxylic acid functionalities or its lack of hydroxyl groups. For CRAMs **2**, **3**, and **4**, the presence of an alkene, a 1,1-dicarboxylic acid functionality, or both of these features likely led to their relative instability within this context. Similarly, **10** remained only in trace quantities, with both its enone functionality and glycosidic linkages likely diminishing its stability. Analogous results are observed in the ASW samples, with the only key differences being the retention of small amounts of CRAM **3** and **4** and the loss of peptide **7**. The loss of **7** is unusual, and while its loss was consistent across all three replicates for ASW (and LW, vide infra), we have no chemical reasoning for this loss and consider it to possibly be an artifact. One speculative explanation for this could be that chloride or bromide radicals are formed during irradiation,^40,41^ which undergo preferential reaction with **7**, but this requires additional experimentation to verify and is not core to the work at hand.

For the irradiation experiment in LW, CRAMs **1** and **3**, and steroid **5** appeared entirely stable, while syringic acid (**8**) underwent significantly less degradation than did the MQ and ASW experiments. CRAMs **2** and **4** were observed in low quantities, while all other compounds were completely removed. The remaining levels of **1**, **3**, **5**, and **8** in LW are considerably higher in comparison to both MQ and ASW and suggest that the DOM from the sampled LW is protecting these compounds from degradation. Possibilities for this include the potential for light-screening by DOM (molecules within DOM acting as quenchers for the tested molecules that could be photochemically excited)^42,43^ or more reactive compounds within the sampled DOM preferentially reacting with any generated reactive oxygen species (ROS) and undergoing modification or mineralization.^21,44^ While this pool of more reactive material from DOM that could quench ROS would eventually be exhausted here, in nature, it would be sporadically replaced by newly leached DOM from catchment soils, depending on the balance of the water residence time of the lake and the amount of sunlight exposure. Assessment of DOM changes from this sample under the same conditions can be found later in Section 3.3.

Examination of the tested compounds by UV–vis spectrophotometry showed that compounds **1**–**5**, **7**, and **11** displayed little or no absorbance that overlapped with the wavelengths of the solar light filter (all UV–visible chromatograms shown in Figure SI9–SI53, page S11–S37). Conversely, compounds **8**–**10** and **12**–**13** absorbed light at wavelengths between 270 and 430 nm. As a potential explanation for the degradation of nonabsorbing molecules, it was considered that **8**–**10** and **12**–**13** could become excited and form reactive intermediates that could immediately react with compounds **1**–**7** and **11** or go on to form other reactive intermediates. Additionally, we wanted to check for the possibility that DMSO or methanol was undergoing photochemical excitation or modification to reactive species by ROS.^45,46^

To investigate various points of contention from the 427 h experiments, a series of shorter tests over 93 h in MQ alone were designed (Figure 3 and Figure SI6, page S8). CRAMs **1**, **2**, and **3** were irradiated individually, as was a mixture of CRAMs **1**, **2**, **3**, and **4**, without additional compounds **5**–**13**, to test whether the addition of compounds **5**–**13** was leading to degradation in MQ. Additionally, two separate experiments were performed on the full mixture of compounds **1**–**13**, one in which no DMSO or methanol was added, and one where much higher concentrations of both DMSO and methanol were added compared to the initial experiments (1 molar *vs*. 2.5 mM methanol, 0.14 mM DMSO from the 427 h experiment). The very high concentration was chosen to provide conditions in which DMSO and methanol could not be consumed by any produced ROS and to check whether their presence was hindering or accelerating degradation. Finally, for all six of these experiments (three single compounds, one CRAM mixture, two compound **1**–**13** mixtures), additional dark control experiments were performed to check for sorption onto glass or potential hydrolysis in water.

For individual irradiation experiments, triacid CRAMs **1** and **2** were only partially degraded, while tetra-acid alkane **3** underwent complete degradation. This aligns with CRAMs **1** and **2** being the only two CRAM compounds (alongside cholic acid (**5**)) remaining in the 427 h irradiation experiment. When all four CRAM compounds were mixed in the absence of compounds **5**–**13**, the results were nearly identical, with triacids **1** and **2** remaining after 93 h of irradiation, while tetra-acids **3** and **4** were fully decomposed. Peaks corresponding to decarboxylated triacid products of compound **3** (i.e., diastereomers of compound **1**) were observed in the single compound experiment, suggesting degradation proceeds via decarboxylation of the more labile 1,1-diacid functionality.

For all dark control experiments, compounds **1**–**5**, **7**–**10**, and **13** were returned at 100% intensity after the 93 h incubation (Figure SI6, page S8), indicating hydrolysis and sorption effects were minimal. Notably, the absence of UV–visible absorbance overlaps of **3** or **4** with the experimental irradiation wavelengths (Figures SI15–SI22, pages S15–S18) suggests that degradation proceeds through some additional excited species. Although trace impurities could be responsible, LC analysis with charged aerosol detection showed all tetra-acid compounds were at least 94% pure,^28^ making extensive degradation unlikely, assuming reaction occurred on a stoichiometric basis. This would require trace impurities at subparts per billion levels to react sequentially and degrade the compounds fully. While the specific mechanism of tetra-acid degradation remains unclear, the identification of decarboxylated products and the lack of degradation in dark controls confirm their instability under the simulated solar irradiation conditions.

For the 93 h incubations comprising all compounds **1**–**13**, the exclusion of methanol and DMSO led to similar results to the 427 h MQ experiments. CRAM **1** and cholic acid (**5**) were the least degraded, CRAM **2** was slightly more degraded than CRAM **1**, and all other compounds that could be detected in the corresponding control samples were entirely degraded. In comparison, the inclusion of high concentrations of methanol and DMSO led to far more extensive degradation of all compounds, including **5**, which was completely unaffected in all 427 h experiments. This, likely, is due to the generation of other reactive species in the irradiation experiments from these cosolvents. While concentrations in the 427 h experiments were low (methanol 2.5 mM, DMSO 0.14 mM), they could still act as additional reactive species until their consumption if ROS were being produced by other photoexcitable molecules. As such, these shorter control experiments suggest that the presence of these cosolvents in the 427 h experiment might result in slightly overestimated degradation relative to comparable environmental conditions. Nevertheless, the trends observed in the 427 h MQ experiments are reinforced by the 93 h experiments in which both methanol and DMSO were excluded.

Further experimentation is ultimately required to determine the mechanisms of degradation for CRAM compounds **2**–**4** under these conditions. This will involve testing the breakdown pathways of these molecules individually. At the environmentally analogous concentrations (20 ppb combined between several stereoisomers) used in these experiments, detection of degradation products by LCMS is difficult. As such, experiments exploring the mechanistic pathways that lead to their breakdown in this setting will require testing at higher concentrations, extraction volumes, or more sensitive equipment but are an important direction for future exploration. This can be supported by degradation experiments using controlled concentrations of ROS that can delineate which transformations are attributable to different reactive species or to direct photolysis. Furthermore, expanding the set of available CRAM-like molecules to include compounds with broader functional group and carbon-backbone diversity will help to examine whether all reduced CRAM-like scaffolds are stable under these irradiation conditions, or whether the decalin (two fused 6-membered rings) structure employed here is part of a subgroup of stable scaffolds.

### 3.2 Biological Incubations

For the biological incubations in both LW and CSW, CRAMs **1**–**4** and tetra-carboxylic acid **13** were largely unaffected by microbial communities. Again, experiments were designed to be as long as was practically feasible to test for stability on the longest possible time scale. Due to challenges in accurate quantification (see Section 2.6 and SI pages S5–S6), we treat any result over 100% as indicating that a compound remains, rather than that compounds of identical mass and retention time have been produced during the experiment. However, establishing this definitively would require further experimentation. In contrast, all other detected compounds were completely removed in community incubation experiments with the exception of **9**, which was observed only in trace quantities in the LW community after 22 days. It is noteworthy that nutrient conditions of the selected waters were on the oligotrophic side (particularly for phosphate); see Section 2.1, but these lower nutrient conditions did not limit the usage of compounds **5** and **7**–**10**, indicating that an active microbial community was able to consume labile compounds in these experiments. It is perhaps unsurprising that compounds **5** and **7**–**10** are degraded in biological incubations; microbial life has evolved a range of mechanisms that utilize natural products as substrates for enzymatic reactions, leading to their degradation or modification.^47–50^ Tripeptides such as **7** are derived from universal amino acid monomers, **9** and **10** contain energy-rich sugar functionalities, **8** is a common plant metabolite, and bile acids such as **5** are readily degraded by soil and water bacteria.^48^

In comparison to the microbial community experiments, few isolated fungal species appeared capable of degrading any of the tested compounds (Figure SI7, page S9). Ultimately, **7** and **9** were reliably degraded by the fungal strains *Rhodotorula sphaerocarpa* and unknown fungal strain ECO1-30, and **10** was partially degraded by *Cladosporium* sp.. Syringic acid (**8**) was not detected in the controls or tests in this data set. While the fungal degradation of bile acids is generally known,^51,52^ and fungi possess cytochrome P450 (CYP) enzymes capable of the oxidation of a wide number of substrates,^53,54^ the strains used here were unable to degrade cholic acid (**5**) in this context, a clear difference from the community experiments. It should be noted that natural microbial communities often collaborate by codegrading organic matter and utilizing each other’s breakdown byproducts.^55^ As the fungal species in this study were incubated individually, this community effect was absent, limiting the diversity of enzymes required for the degradation of the complex material. However, the inability for both environmental microbial communities and isolated fungal strains to degrade CRAM-like analogues **1**–**4** or tetra-carboxylic acid **13** provides valuable evidence for their biological recalcitrance. Ultimately, CRAMs **1**–**4** represent only a small portion of the scaffolds and functional group compositions that make up natural CRAM, and as such, a broader range of compounds, as well as additional microbes, must be tested to determine the extent of environmental CRAM biological recalcitrance.

### 3.3 Experimental Effects on Lake DOM

In the lake water experiments (i.e., Sol.LW, LW22 and LW259), the lake water used as a matrix for study of compound stability contained thousands of molecular formulas from DOM in the sample. The identities of the compounds making up these molecular formulas are unknown, as explained in the introduction, and each molecular formula is constituted by an unknown number of structural isomers. Despite the lack of knowledge of the chemical structures making up DOM, it is possible to measure changes to the mixture, at least to the portion that is ionizable to deprotonated ions by electrospray, using direct infusion HRMS or LC-HRMS. Using the data already in hand from LC-HRMS analysis of compounds **1**–**13**, we assessed overall “molecular formula level” changes to the DOM mixture in the triplicate samples at experimental conditions LW-control (time zero), Sol.LW, and LW22, as these were measured in the same analytical run.

Assessment of the lake water DOM changes after experiments showed minor but statistically significant changes (Student’s *t* test on whole sample metrics) in the irradiation experiment (Sol.LW), and essentially no detectable changes in the biological degradation experiment (LW22), in line with recent findings regarding biodegradability of SPE-DOM by ESI-MS from the same lake (Table 1).^56^ In addition to broad metric changes to average H/C and molecular mass, Bray–Curtis dissimilarity was evaluated based on all normalized mass spectral peak intensities (Figure 5b), which allowed preparation of a principal coordinate diagram based on dissimilarity (distances; Figure 5c). This showed that most (69%) of the variability in the whole data set could be explained by one coordinate that separated the irradiated samples from the controls and incubation samples.

**Table 1:** Weighted Average Metrics from the First Lake Water Experiments^a^

sample	O/C_wa_	H/C_wa_	*m*/*z*_wa_	number of formulas
control LW	0.488 ± 0.005	1.289 ± 0.006	359 ± 3	4041 ± 130
LW22	0.482 ± 0.003	1.292 ± 0.007	354 ± 3	4252 ± 143
Sol.LW	0.494 ± 0.004	**1.318 ± 0.006**	344 ± 7	4224 ± 144

a Shown are average oxygen/carbon ratio (O/C), hydrogen to carbon ratio (H/C), mass to charge ratio (m/z), and number of peaks detected, as intensity-weighted averages, with standard deviations shown (*n* = 3). Results that are statistically significantly different from the control (Student’s *t* test) are shown in bold.

**Figure 5:**
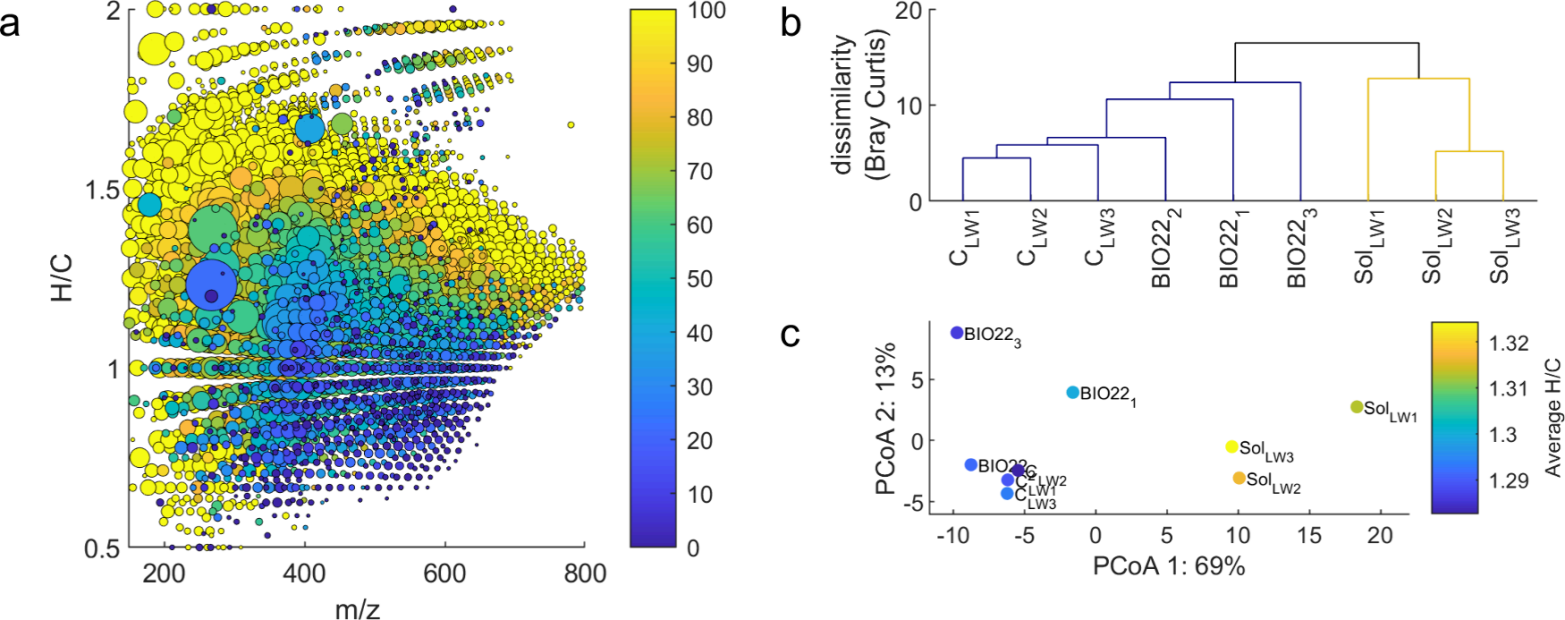
(a) Compounds detected in lake water DOM, colored according to relative loss in the longer irradiation experiment (Sol.LW). Each compound is plotted at the determined H/C ratio vs m/z, and all compounds detected in the lake water control sample are shown. Point size is shown according to square root of intensity (mean, *n* = 3), for scaling purposes (i.e., in order to see more peaks). Color is shown according to the difference in relative intensity between the irradiated and control sample (irradiated/control × 100), where initial values were normalized per sample to sum to 100. This means that the difference is not quantitative (i.e., showing % loss), but rather qualitative, showing extent of change relative to other peaks in the sample. The color scale is limited to 100 to focus on the peaks that had a relative loss of intensity, and the points were plotted in decreasing color order to highlight the peaks that were lost. (b) Bray–Curtis dissimilarity-based cluster dendrogram with cutoff set to 15% dissimilarity. (c) Principal coordinate analysis (PCoA) based on a Bray–Curtis dissimilarity matrix, showing scores in the first two dimensions (totaling 82% of data variability), with H/C_wa_ indicated by color.

The lack of apparent change in the biological incubations is partly due to the limitations of combining SPE and negative-mode ESI to measure the hydrophilic, labile species, rather than due to lack of degradation of total DOC. The changes found to the more hydrophobic part of the DOM mixture in the irradiation experiment were in line with expectations, with higher-molecular-weight unsaturated compounds being the most sensitive to loss (Figure 5).

It is noteworthy that the results obtained for the lake water DOM match fairly closely to those obtained for the synthesized CRAM compounds and aromatic carboxylic acid-rich compound **13**, specifically that the DOM molecular peaks were partially degraded in the irradiation experiment but largely stable to the biological incubation. This is by no means proof that DOM is composed of these types of compounds but highlights the utility of these compounds as control compounds in DOM degradation experiments.

### 3.4 Research Outcomes and Future Directions

In the irradiation experiments, tests probing the physical and chemical stabilities of our initial CRAM analogues **1**–**4** within a small curated chemical library highlighted that specific chemical functionalities were the strongest indicator of stability. CRAM **1** and cholic acid (**5**) were the only compounds that remained in significant quantities after irradiation. The majority of compounds that contained 1,1-diacid, enone, and aromatic functionalities were either completely degraded or remained only in trace quantities in all experiments, and the presence of DOM only partially protected some of those same compounds. As an exception, CRAM-like compound **3**, which contained a 1,1-diacid, and had a fully reduced backbone remained unaffected after irradiation in the presence of DOM, as did triacid CRAM **1** with its fully reduced carbon backbone. Notably, CRAM alkenes **2** and **4** were not protected to the same extent by lake water DOM. Future experiments will probe the mechanisms through which DOM protects alkane CRAM-like compounds by assessing the extent to which it screens light, preferentially reacts with ROS, and whether it can directly quench photoexcited compounds with molecular compositions relevant to DOM. Furthermore, select compounds will have their breakdown products tracked, to examine potential degradation pathways for compounds with CRAM-like chemical formulas in the environment, while diastereomeric mixtures will be purified into single isomers to test the effects of relative stereochemistry under the same conditions.

Within the set of biological experiments, a compound’s existence as a direct biological metabolite was the best indicator of its stability to challenges by either microbial communities or isolated fungal strains. It is notable that no member of either microbial community was able to harness any of the CRAM analogues **1**–**4** or tetra-carboxylic acid **13** over approximately 8 months under warm conditions (20 °C). This, of course, could be simply due to the fact that these compounds are not known biological substrates. Similarly, it is likely that a significant portion of recalcitrant DOM is modified by abiotic processes,^3^ being derived from the geochemical degradation of biological compounds through reaction with light and ROS.^57^ Thus, the stability of these synthesized compounds in this context is in part indicative of the broader recalcitrance to biological challenges of geochemically processed compounds with CRAM-like functionalities and molecular formulas. Further investigation of biological recalcitrance will focus predominantly on the diversification of CRAM-analogue scaffolds to test whether specific carbon backbones may render these compounds biologically available. Additionally, tests tailored toward the aggregation and complexation of these compounds are envisioned to expand this initial set of recalcitrance experiments upon isolated CRAM-like compounds.

A key problem in understanding the chemical properties of DOM is understanding how theorized chemical classes like CRAM behave. In general, it is taken for granted that these species exist, and it is generally ignored how little experimental data exist for isolated compounds with structural features accurate to those of theorized CRAM. As a result, much is written about their environmental behavior based on top–down correlations, where molecular formulas or CHO ratios are ascribed to a sole chemical class. This approach, while useful in some settings, is fundamentally flawed when trying to describe chemical composition or reactivity, as isomerism is poorly understood and the transformation of individual molecular formulas cannot be tracked in a matrix as complex as DOM. Here, we have taken the opposite approach, where CRAM-like compounds with appropriate chemical functionality have been placed into simulated environmental contexts and studied for their stability. This work is the first step in verifying assumptions that are made about the chemistry of CRAM. While the studies here represent the beginning of a long-term research direction, they provide previously inaccessible and concrete data showing the stability of small CRAM-like molecules to both photochemical and biological challenges.

## Supplementary Material





## Notes

Additional experimental details, including materials, sample preparation, experimental setup, data analysis, quality control, and experimental considerations (PDF)
Processing steps to convert feature data from mzMine to the graphs found in the paper according to eqs 1–3 (XLSX)

## References

[1] Hansell D. A. (2013). Recalcitrant
Dissolved Organic Carbon Fractions. Annu. Rev.
Mar. Sci..

[2] Dittmar T., Lennartz S. T., Buck-Wiese H., Hansell D. A., Santinelli C., Vanni C., Blasius B., Hehemann J.-H. (2021). Enigmatic Persistence
of Dissolved Organic Matter in the Ocean. Nat.
Earth. Rev. Environ..

[3] Dittmar, T. Chapter 7 - Reasons Behind the Long-Term Stability of Dissolved Organic Matter. In Biogeochemistry of Marine Dissolved Organic Matter (2nd ed.); Elsevier: 2014; pp 369–388.

[4] Jiao N., Robinson C., Azam F., Thomas H., Baltar F., Dang H., Hardman-Mountford N.
J., Johnson M., Kirchman D. L., Koch B. P., Legendre L., Li C., Liu J., Luo T., Luo Y.-W., Mitra A., Romanou A., Tang K., Wang X., Zhang C., Zhang R. (2014). Mechanisms
of Microbial Carbon Sequestration in the Ocean - Future Research Directions. Biogeosciences.

[5] Arrieta J. M., Mayol E., Hansman R. L., Herndl G. J., Dittmar T., Duarte C. M. (2015). Dilution Limits
Dissolved Organic Carbon Utilization
in the Deep Ocean. Science.

[6] Kritzberg E. S., Arrieta J. M., Duarte C. M. (2010). Temperature
and Phosphorus Regulating
Carbon Flux through Bacteria in a Coastal Marine System. Aquat. Microb. Ecol..

[7] Thingstad T. F., Krom M. D., Mantoura R. F. C., Flaten G. A. F., Groom S., Herut B., Kress N., Law C. S., Pasternak A., Pitta P., Psarra S., Rassoulzadegan F., Tanaka T., Tselepides A., Wassmann P., Woodward E. M. S., Riser C. W., Zodiatis G., Zohary T. (2005). Nature of Phosphorus
Limitation in the Ultraoligotrophic Eastern Mediterranean. Science.

[8] Mopper, K. ; Kieber, D. J. ; Stubbins, A. Chapter 8 - Marine Photochemistry of Organic Matter: Processes and Impacts. In Biogeochemistry of Marine Dissolved Organic Matter (2nd ed.); Elsevier: 2014; pp 389–450.

[9] Wetzel R. G., Hatcher P. G., Bianchi T. S. (1995). Natural
Photolysis by Ultraviolet
Irradiance of Recalcitrant Dissolved Organic Matter to Simple Substrates
for Rapidbacterial Metabolism. Limnol. Oceanogr..

[10] Tranvik L., Kokalj S. (1998). Decreased Biodegradability of Algal DOC Due to Interactive
Effects of UV Radiation and Humic Matter. Aquat.
Microb. Ecol..

[11] Cao F., Zhu Y., Kieber D. J., Miller W. L. (2020). Distribution and Photo-Reactivity
of Chromophoric and Fluorescent Dissolved Organic Matter in the Northeastern
North Pacific Ocean. Deep Sea Res. Pt. I.

[12] Riedel T., Zark M., Vähätalo A. V., Niggemann J., Spencer R. G., Hernes P. J., Dittmar T. (2016). Molecular
Signatures
of Biogeochemical Transformations in Dissolved Organic Matter from
Ten World Rivers. Front. Earth Sci..

[13] Koch B., Kattner G., Witt M., Passow U. (2014). Molecular
Insights
into the Microbial Formation of Marine Dissolved Organic Matter: Recalcitrant
or Labile?. Biogeosciences.

[14] Kujawinski E. B. (2011). The Impact
of Microbial Metabolism on Marine Dissolved Organic Matter. Annu. Rev. Mar. Sci..

[15] Hur J., Lee B.-M., Shin H.-S. (2011). Microbial
Degradation of Dissolved
Organic Matter (DOM) and Its Influence on Phenanthrene–DOM
Interactions. Chemosphere.

[16] Carlson, C. A. ; Hansell, D. A. Chapter 3 - DOM Sources, Sinks, Reactivity, and Budgets. In Biogeochemistry of Marine Dissolved Organic Matter; Hansell, D. A. , Carlson, C. A. , Eds.; Elsevier: 2015; pp 65–126.

[17] Hawkes J. A., Rossel P. E., Stubbins A., Butterfield D., Connelly D. P., Achterberg E. P., Koschinsky A., Chavagnac V., Hansen C. T., Bach W., Dittmar T. (2015). Efficient
Removal of Recalcitrant Deep-Ocean Dissolved Organic Matter during
Hydrothermal Circulation. Nat. Geosci..

[18] Coppola A. I., Ziolkowski L. A., Masiello C. A., Druffel E. R. (2014). Aged Black Carbon
in Marine Sediments and Sinking Particles. Geophys.
Res. Lett..

[19] Dunne J. P., Sarmiento J. L., Gnanadesikan A. (2007). A Synthesis of Global Particle Export
from the Surface Ocean and Cycling through the Ocean Interior and
on the Seafloor. Global Biogeochem. Cycles.

[20] Liu Z., Cai R., Chen Y. L., Zhuo X., He C., Zheng Q., He D., Shi Q., Jiao N., Gralnick J. A. (2023). Direct Production
of Bio-Recalcitrant Carboxyl-Rich Alicyclic Molecules Evidenced in
a Bacterium-Induced Steroid Degradation Experiment. Microbiol. Spectrum.

[21] Catalá T. S., Rossel P. E., Álvarez-Gómez F., Tebben J., Figueroa F. L., Dittmar T. (2020). Antioxidant Activity
and Phenolic Content of Marine Dissolved Organic Matter and Their
Relation to Molecular Composition. Front. Mar.
Sci..

[22] Maizel A. C., Remucal C. K. (2017). The Effect of Advanced
Secondary Municipal Wastewater
Treatment on the Molecular Composition of Dissolved Organic Matter. Water Res..

[23] Geng C.-X., Cao N., Xu W., He C., Yuan Z.-W., Liu J.-W., Shi Q., Xu C.-M., Liu S.-T., Zhao H.-Z. (2018). Molecular Characterization
of Organics Removed by a Covalently Bound Inorganic-Organic Hybrid
Coagulant for Advanced Treatment of Municipal Sewage. Environ. Sci. Technol..

[24] Chen W., Zhuo X., He C., Shi Q., Li Q. (2020). Molecular
Investigation into the Transformation of Dissolved Organic Matter
in Mature Landfill Leachate during Treatment in a Combined Membrane
Bioreactor-Reverse Osmosis Process. J. Hazard.
Mater..

[25] Mesfioui R., Love N. G., Bronk D. A., Mulholland M. R., Hatcher P. G. (2012). Reactivity and Chemical Characterization of Effluent
Organic Nitrogen from Wastewater Treatment Plants Determined by Fourier
Transform Ion Cyclotron Resonance Mass Spectrometry. Water Res..

[26] Wang Y., Zhang Z., Han L., Sun K., Jin J., Yang Y., Yang Y., Hao Z., Liu J., Xing B. (2019). Preferential Molecular Fractionation of Dissolved Organic Matter
by Iron Minerals with Different Oxidation States. Chem. Geol..

[27] Mitschke N., Vemulapalli S., Dittmar T. (2023). NMR Spectroscopy of Dissolved Organic
Matter: A Review. Environ. Chem. Lett..

[28] Craig A. J., Moodie L. W., Hawkes J. A. (2024). Preparation of Simple Bicyclic Carboxylate-Rich
Alicyclic Molecules for the Investigation of Dissolved Organic Matter. Environ. Sci. Technol..

[29] Hertkorn N., Benner R., Frommberger M., Schmitt-Kopplin P., Witt M., Kaiser K., Kettrup A., Hedges J. I. (2006). Characterization
of a Major Refractory Component of Marine Dissolved Organic Matter. Geochim. Cosmochim. Acta.

[30] Witt M., Fuchser J., Koch B. P. (2009). Fragmentation
Studies of Fulvic Acids
Using Collision Induced Dissociation Fourier Transform Ion Cyclotron
Resonance Mass Spectrometry. Anal. Chem..

[31] Hawkes J.
A., Patriarca C., Sjöberg P. J., Tranvik L. J., Bergquist J. (2018). Extreme Isomeric
Complexity of Dissolved Organic Matter Found across Aquatic Environments. Limnol. Oceanogr. Lett..

[32] Hawkes J. A., Sjöberg P. J., Bergquist J., Tranvik L. (2019). Complexity of Dissolved
Organic Matter in the Molecular Size Dimension: Insights from Coupled
Size Exclusion Chromatography Electrospray Ionisation Mass Spectrometry. Faraday Discuss..

[33] Han L., Kaesler J., Peng C., Reemtsma T., Lechtenfeld O. J. (2021). Online
Counter Gradient LC-FT-ICR-MS Enables Detection of Highly Polar Natural
Organic Matter Fractions. Anal. Chem..

[34] Matos R. R., Jennings E. K., Kaesler J., Reemtsma T., Koch B. P., Lechtenfeld O. J. (2024). Post Column Infusion of an Internal Standard into LC-FT-ICR
MS Enables Semi-Quantitative Comparison of Dissolved Organic Matter
in Original Samples. Analyst.

[35] Kester D. R., Duedall I. W., Connors D. N., Pytkowicz R. M. (1967). Preparation
of Artificial Seawater 1. Limnol. Oceanogr..

[36] Environmental data MVM, A web service with land, water, and environmental data. http://miljodata.slu.se/mvm/ (accessed 2025–02–03).

[37] Swedish Ocean Archive. https://shark.smhi.se/hamta-data/ (accessed 2025–02–03).

[38] Breyer E., Espada-Hinojosa S., Reitbauer M., Karunarathna S. C., Baltar F. (2023). Physiological Properties of Three Pelagic Fungi Isolated
from the Atlantic Ocean. J. Fungus.

[39] Jäger S., Winkler K., Pfüller U., Scheffler A. (2007). Solubility
Studies of Oleanolic Acid and Betulinic Acid in Aqueous Solutions
and Plant Extracts of Viscum Album L. Planta
Med..

[40] Zhang K., Parker K. M. (2018). Halogen Radical Oxidants in Natural and Engineered
Aquatic Systems. Environ. Sci. Technol..

[41] Parker K. M., Mitch W. A. (2016). Halogen Radicals Contribute to Photooxidation in Coastal
and Estuarine Waters. Proc. Natl. Acad. Sci.
U. S. A..

[42] Janssen E. M.-L., Erickson P. R., McNeill K. (2014). Dual Roles of Dissolved Organic Matter
as Sensitizer and Quencher in the Photooxidation of Tryptophan. Environ. Sci. Technol..

[43] Baker A. (2005). Thermal Fluorescence
Quenching Properties of Dissolved Organic Matter. Water Res..

[44] Romera-Castillo C., Jaffé R. (2015). Free Radical Scavenging (Antioxidant
Activity) of Natural
Dissolved Organic Matter. Mar. Chem..

[45] Zhou X., Mopper K. (1990). Determination of Photochemically
Produced Hydroxyl
Radicals in Seawater and Freshwater. Mar. Chem..

[46] Vaughan P. P., Blough N. V. (1998). Photochemical Formation
of Hydroxyl Radical by Constituents
of Natural Waters. Environ. Sci. Technol..

[47] Sedlaczek L., Smith L. L. (1988). Biotransformations
of Steroids. Crit. Rev. Biotechnol..

[48] Feller F. M., Holert J., Yücel O., Philipp B. (2021). Degradation of Bile
Acids by Soil and Water Bacteria. Microorganisms.

[49] Rehms H., Barz W. (1995). Degradation of Stachyose,
Raffinose, Melibiose and Sucrose by Different
Tempe-Producing Rhizopus Fungi. Appl. Microbiol.
Biotechnol..

[50] Phelps C., Young L. (1997). Microbial Metabolism
of the Plant Phenolic Compounds Ferulic and
Syringic Acids under Three Anaerobic Conditions. Microb. Ecol..

[51] Wei X., Yao C., He X., Li J., Wang Y., Wang C., Chen Q., Ma X., Guo D. (2024). Biotransformation of
Chenodeoxycholic Acid by Human Intestinal Fungi and the Agonistic
Effects on FXR. Phytochemistry.

[52] Yang B., Zha R., Zhao W., Gong D., Meng X., Zhang Z., Zhu L., Qi N., Wang B. (2021). Comparative Transcriptome Analysis
of the Fungus Gibberella Zeae Transforming Lithocholic Acid into Ursodeoxycholic
Acid. Biotechnol. Lett..

[53] Durairaj P., Hur J. S., Yun H. (2016). Versatile
Biocatalysis of Fungal
Cytochrome P450 Monooxygenases. Microb. Cell
Fact..

[54] Črešnar B., Petrič Š. (2011). Cytochrome
P450 Enzymes in the Fungal Kingdom. Biochim.
Biophys. Acta - Proteins and Proteomics.

[55] Frey-Klett P., Burlinson P., Deveau A., Barret M., Tarkka M., Sarniguet A. (2011). Bacterial-Fungal
Interactions: Hyphens between Agricultural,
Clinical, Environmental, and Food Microbiologists. Microbiol. Mol. Bio. Rev..

[56] Grasset C., Groeneveld M., Tranvik L. J., Robertson L. P., Hawkes J. A. (2023). Hydrophilic Species
Are the Most Biodegradable Components
of Freshwater Dissolved Organic Matter. Environ.
Sci. Technol..

[57] Stubbins A., Dittmar T. (2015). Illuminating the Deep: Molecular Signatures of Photochemical
Alteration of Dissolved Organic Matter from North Atlantic Deep Water. Mar. Chem..

[58] Craig, A. ; Norouzi, M. ; Löffler, P. ; Lai, F. Y. ; Mtibaà, R. ; Breyer, E. ; Baltar, F. ; Moodie, L. W. K. ; Hawkes, J. A. Investigating the Stability of Individual Carboxylate Rich Alicyclic Molecules Under Simulated Environmental Irradiation and Microbial Incubation Conditions. ChemRxiv, 2025, 10.26434/chemrxiv-2025-3ndrr. PMC12392460 40808488

