# Supplementary material for: Investigating the Stability of Individual Carboxylate-Rich Alicyclic Molecules Under Simulated Environmental Irradiation and Microbial Incubation Conditions
Source: Environ Sci Technol. 2025 Aug 14;59(33):17571–80. doi: 10.1021/acs.est.5c01958 (PMC12392460; doi:10.1021/acs.est.5c01958)
Supplement: Supplementary file 1 [file es5c01958_si_001.pdf]

Supporting Information

Investigating the Stability of Individual Carboxylate Rich Alicyclic Molecules Under Simulated Environmental Irradiation and Microbial Incubation Conditions

Alexander J. Craig,[1,2] Mahsa Norouzi,[1] Paul Löffler,[3] Foon Yin Lai,[3] Rim Mtibaà,[4] Eva Breyer,[4,5] Federico Baltar,[4,5] Lindon W. K. Moodie,[2] Jeffrey A. Hawkes[1]

[1]Analytical Chemistry, Department of Chemistry BMC, Uppsala University, Uppsala 752 37, Sweden.

[2]Drug Design and Discovery, Department of Medicinal Chemistry, Uppsala University, Uppsala 752 37, Sweden

[3]Swedish University of Agricultural Sciences, Department of Aquatic Sciences and Assessment, Box 7050, 750 07 Uppsala, Sweden

[4] Fungal & Biogeochemical Oceanography Group, Department of Functional and Evolutionary Ecology, University of Vienna, Vienna, Austria

[5] Fungal & Biogeochemical Oceanography Group, College of Oceanography and Ecological Science, Shanghai Ocean University, Shanghai, China

Summary: 38 pages, 53 figures

Contents:

Experimental Details	**S2-S10**
UV-visible absorbance spectra	**S11-S37**
References	**S38**

Materials

Compounds were purchased from TCI Europe and Sigma Aldrich, or synthesized.[1] Acetonitrile (LiChroSolv LCMS grade), methanol (LiChroSolv LCMS grade), and water (LiChroSolv LCMS grade), were purchased from VWR Sweden. Four sample matrices were used in the experiments, referred to as MQ (MilliQ water), ASW (Artificial seawater),[2] LW (surface lake water taken from Långsjö, near Bjorklinge, Sweden: 60°02'31.97" N, 17°33'36.40" E on August 30th 2023 and filtered through ashed glass fiber filter (GF/F)), and CSW (Coastal seawater, taken from the jetty at Tjärnö Marine Laboratory, Sweden on 5th September, 2023 and filtered through ashed GF/F.

Preparation of compound mixture and control samples

Each of the 17 compounds was prepared to 0.1 mg L^{-1} (first experiment set) or 1 mg L^{-1} (second set) in dimethyl sulfoxide. These were combined into a single stock with 5 ppm of each compound, and were diluted using 50% methanol. This stock was added at 0.2 mL per litre of sample (MQ, ASW, GF/F Filtered Lake Water (LW) and GF/F Filtered coastal seawater (CSW)) to achieve a starting concentration of 10 ppb. 20 mL of each spiked sample type (samples with added compounds) were directly acidified and extracted onto Agilent PPL sorbent (100 mg) as a time=0 control, in triplicate. Additionally, a non-spiked lake sample (referred to as Lake-Zero) was extracted as a singlet. At the time of the second set of experimental extractions (about 250 days later), a second set of spiked lake water triplicates, and a second 'lake-zero' sample were also extracted, and used as controls for those samples.

Sample treatments and extractions

In all sample treatments, 40 mL of spiked sample was used.

Solar irradiation experiment

The irradiation experiment was carried out using in an Atlas Suntest XXL+FD, which was equipped with three xenon lamps and an Atlas Suntest Daylight filter. Samples were placed into 50 mL beakers before being covered with a watch glass, both made from borosilicate 3.3 to ensure maximal light transparency, and sealed with parafilm. The samples were irradiated for a total time of 427 hr with a continuous irradiation intensity of 65 W m^{-2} (adjusted over 300-400 nm). Light intensity was regularly cross-checked using a SP-110 (SolData Instruments, Silkeborg, Denmark). The temperature remained stable at 25 °C over the entire period. The cumulative irradiance exposure over the course of the experiment was 10000 kJ m^{-2}. Assuming a typical cumulative irradiance of 1.75 W m^2 at sea level[3] with 12 h of sunlight per day, this corresponds to approximately 75.6 kJ m^{-2}, making our exposure 132 more intense than average sea level conditions. Notably, two samples, SOL_MQ_1 and SOL_ASW_3, dried out significantly during the experiment, resulting in low analyte concentrations. These samples were excluded from the final analysis, ensuring only duplicate samples were used for statistical evaluation.

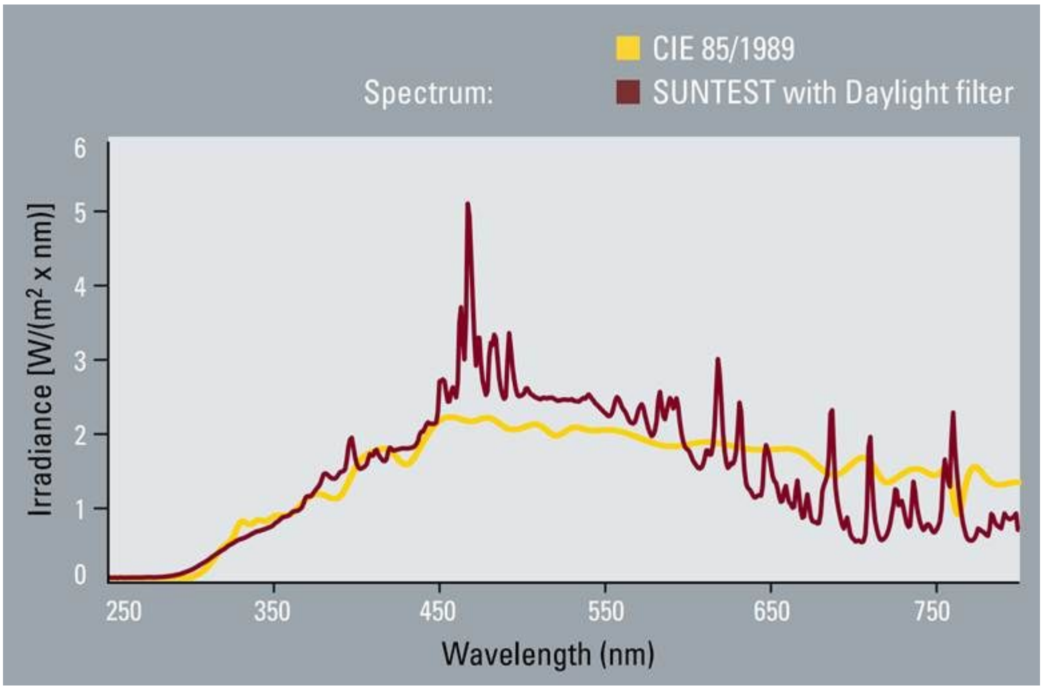

Figure SI1: *Irradiance spectrum of the Suntest XXL+FD device (red) compared with the Commission Internationale de l'Eclairage standard of solar irradiation on Earth,[4] showing particularly good alignment at UV wavelengths. Reproduced from the SUNTEST Family Brochure – English – 2024 – 02, https://www.atlas-mts.com/products/standard-instruments/xenon-arc-weathering-test/suntest/xxl-fd.*

Biological degradation experiment

In order to inoculate the Lake sample with a natural microbial community, 2% sample volume of unfiltered lake water was added into the GF/F filtered samples. Six samples (40 mL) were prepared inside closed 100 mL Schott bottles (i.e. >60 mL headspace), and these were placed at 20 °C in a dark, temperature-controlled room, and left stationary. Three of these were extracted after 22 days (LW22), and three were extracted after 259 days (LW259). On the 8th September 2023, GF/F Filtered CSW experiments were initiated (also with 2% volume unfiltered CSW added), and these were extracted after 251 days (CSW251).

Marine fungi degradation experiment

Four marine fungal cultures (*Rhodotorula sphaerocarpa, unknown fungal strain ECO1-30, Cladosporium sp., and Sakaguchia dacryoidea* were used.[5] All investigated fungi were originally isolated from the marine pelagic water column. Fungal isolates were maintained on a solid nutrient-rich medium solidified with 1.5% agar (pH 8). The composition of the medium was (g L^{-1}): agar 15, glucose 10, peptone 5, yeast extract 3, malt extract 3, artificial sea salts 35, and 0.2 chloramphenicol.[5] All plates were incubated at room temperature in the dark. Fresh fungal material was obtained by growing the isolates in a liquid nutrient-rich medium with pH 8.0 (same composition mentioned above).[5] After a few days of incubation at room temperature, the fungal biomass was crushed, washed several times, and stored at 4 °C in a synthetic mineral salt medium with 10 g L^{-1} NaCl for carbon starvation.

Artificial seawater medium supplemented with compound mixture, as the sole carbon source, was used for degradation experiments. The artificial seawater was prepared according to the standard ASTM 1141–98 with some modifications, as shown in Table 1. The medium was supplemented with

sodium phosphate (NH_2PO_4) and ammonium chloride (NH_4Cl) with a final concentration of 10 µM and 1 mM, respectively. Fungal cultures were performed in 250 mL Erlenmeyer flasks containing 50 mL of artificial seawater (pH 8.0) and inoculated with 1% fresh fungal suspension. Compounds **1**-**4** were added at a final concentration of 0.02 mg L^{-1} per compound, and compounds **5**-**13** were added at a final concentration of 0.01 mg L^{-1} per compound. The flasks were incubated on a shaker incubator (Lab Companion, Shaking Incubator model ISS-7100R, 120 rpm, Billerica, MA, USA) at 20 °C in the dark. Abiotic controls were carried out in an uninoculated medium under the same experimental conditions. All assays were carried out in triplicates.

After 102 days of incubation, cultures were subjected to solid phase extraction (SPE) to extract and quantify the remaining CRAMs and metabolites. Therefore, the entire cultures were acidified to pH 2.0 and transferred to sterile conical tubes to determine the initial weight before the extraction. Afterward, the fungal biomass was separated from the supernatant by centrifugation at 3000 x g and 20 °C for 30 min. The PPL columns were conditioned sequentially with 3 mL methanol and 3 mL acidified Milli-Q water (pH 2). The centrifuged samples were loaded onto the columns, which were then washed sequentially with 3 mL acidified Milli-Q water (pH 2). The columns were dried for more than 60 min under N_2 vacuum.

These dried columns were received at the Uppsala University analytical chemistry department on 16th April 2024. They were stored in the fridge and then dried and eluted with 1.5 mL methanol on 15th May 2024. The extracts were dried entirely using a vacuum centrifuge at 40 °C, and were then dissolved in 200 µL 5% acetonitrile containing 1ppm hippuric acid as an internal standard. Most of these samples contained a solid residue at this stage, so they were centrifuged in Eppendorf vials and 60 µL of the supernatant was pipetted out for analysis.

Table SI1: *Chemical composition of the modified seawater and saline solutions concentration (g* L^{-1}*).*

Compound	Seawater ASTM	Solution 1 (Anhydrous salts)	Solution 2 (Hydrous salts)
NaCl	24.53	24.53	–
Na_2SO_4	4.09	4.09	–
KCl	0.695	0.7	–
$NaHCO_3$	0.201	0.2	–
KBr	0.101	0.1	–
H_3BO_3	0.027	0.003	–
NaF	0.003	0.003	–
$MgCl_2$	5.20	–	11.10
$CaCl_2$	1.16	–	1.54

Compound	Seawater ASTM	Solution 1 (Anhydrous salts)	Solution 2 (Hydrous salts)
$SrCl_2$	0.025	–	0.017

Sample Extraction

Samples were acidified and extracted (either 20 or 40 mL, weighed accurately), using Agilent PPL. After rinsing with 0.1% HCl and drying with nitrogen gas, compounds were eluted from the cartridges (100 mg PPL) using 1.5 mL LCMS grade methanol, and this was dried down entirely in a vacuum centrifuge. The samples were re-dissolved with 0.2 mL 5% acetonitrile containing 1 ppm hippuric acid as an internal standard.

Sample Analysis

Samples were analysed using ultra performance liquid chromatography coupled to electrospray ionization high resolution mass spectrometry (UPLC-ESI-HRMS), using a Vanquish UPLC (Thermo Fisher) and an Orbitrap Velos LTQ Pro (Thermo Fisher). The mobile phases for chromatography were 0.1% formic acid in MilliQ water and 0.1% formic acid in LCMS-grade acetonitrile, and these were used in a gradient method at 400 µL min^{-1}, starting at 5% B for 1 minute, ramping to 95% B at 10 minutes, isocratic for 1 minute and then returning to 5% B until 15 minutes. The column was a Phenomenex Kinetex C18 column (2.1 × 150 mm, 1.7 µm) at a flow rate of 0.4 mL min^{-1}, set to 50 °C to decrease back pressure. Samples were injected at 10 µL. Electrospray was conducted in negative mode at -3.5 kV with Sheath gas set to 25, auxiliary gas set to 5, heater set to 200 °C and inlet capillary set to 300 °C. The S-lens was tuned to 68.8%, AGC target was 1e6 and the maximum ion trapping time was 50 ms. The transient range was from 150-1000 Da, and resolution was set to 60,000 in order to decrease duty cycle, which was typically about 1.67 s.

LCMS data processing for lake water DOM peak assignment

Formulas were assigned to lake DOM samples using an in-house MATLAB routine. All transients had masses assigned after mass calibration with hippuric acid at the apex intensity of this XIC. Formulas were assigned between m/z 150-800, with constraints: H/C 0.3 - 2, O/C ≤ 1, N ≤ 1, even electron ions, double bond equivalents minus oxygen ≤ 10, mass defect 0.3-0.9. Noise was determined in each transient as the 95th percentile of peaks with mass defect 0.7-0.8, and peaks were only considered for assignment if they passed 1.5x this level. Formulas were assigned as the closest (usually only) match < 2 ppm. After assignment, all intensities were summed across the whole chromatogram to give a single peak list with intensities. Three blank samples were used to determine blank peaks, and peaks present in other samples had to be 10x higher than the average blank, or their intensities were changed to zero. The resulting data was averaged per sample type and then normalized to sum to 100 for each mean sample for comparison between samples.

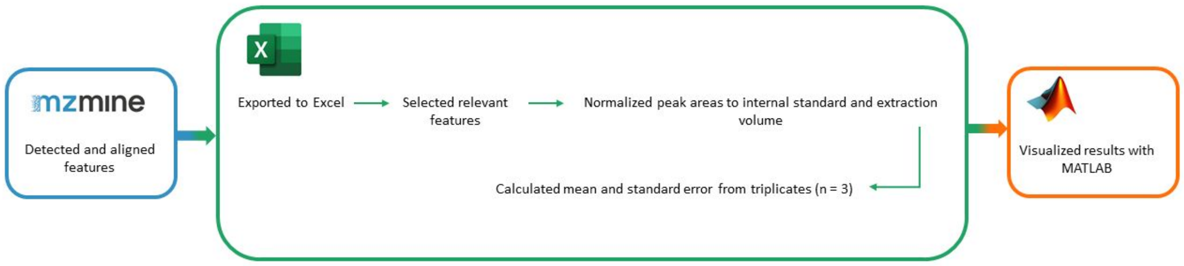

Figure SI2*: Workflow for LC-MS data processing: mzXML files are imported into MZmine for feature detection and alignment, exported to Excel for feature selection, normalized to an internal standard (hippuric acid) and extraction volume, and finally analyzed (mean ± SD of triplicates) and visualized in MATLAB.*

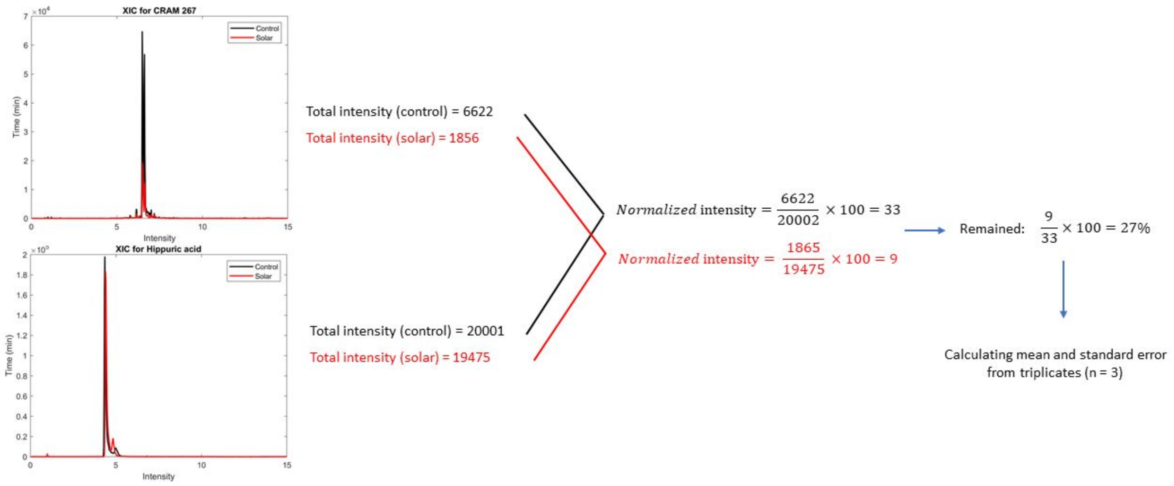

Figure SI3: *Example data-processing workflow showing extracted ion chromatograms (XICs) for a single analyte (CRAM 2, top) and the internal standard (hippuric acid, bottom) in control (black) and solar (red) samples.*

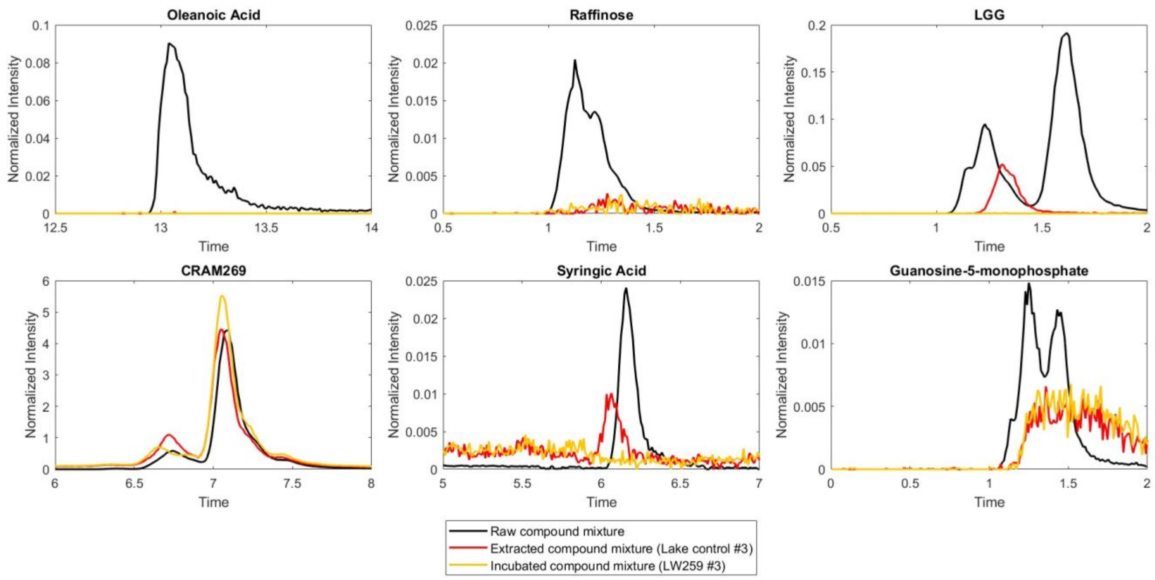

Figure SI4: *Extracted Ion Chromatograms for six selected compounds, three selected samples in each case: raw compound mixture, extracted compound mixture from Lake Control #3, Incubated compound mixture in lake water, 259 days (gold). The following are noteworthy: 1) Oleanolic acid, raffinose and Guanosine-5-monophosphate are not reliably quantifiable or present at all after extraction, and so were not tested for stability. 2) Leu-Gly-Gly has a low extraction efficiency, but is measurable (first peak of two peaks in compound mixture, second is unknown), and this compound removed in the incubation LW259 experiment. 3) CRAM269 (CRAM1 in the manuscript) is effectively extracted and not removed in the incubation LW259 experiment, and has two resolved isomers. Note that the retention time can shift between samples, and in the data processing, and all measurable features (i.e. peaks) are summed in the data processing routine, meaning that retention time alignment is not required.*

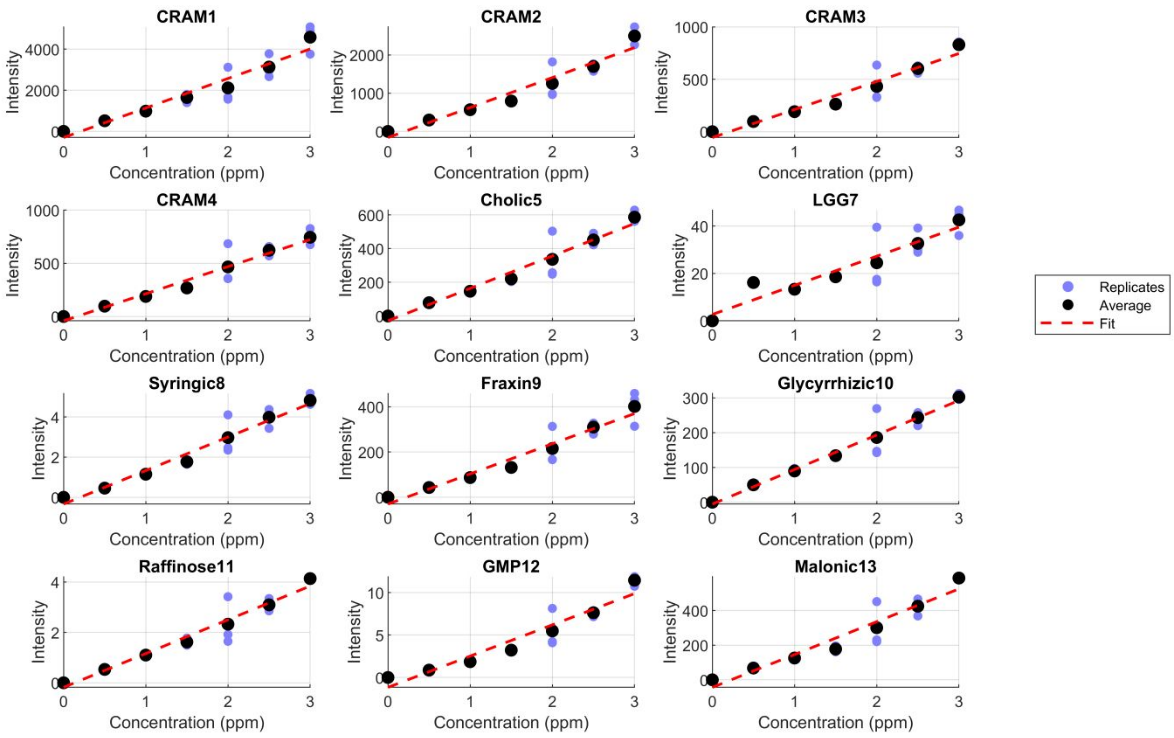

Figure SI5: *Calibration curves for intensity data of the compounds used in the study after normalization to 1 ppm Hippuric acid standard, showing reasonable linearity past the 2 ppm value used for control concentrations (after extraction and concentration), allowing semi-quantification at the level used in the study.*

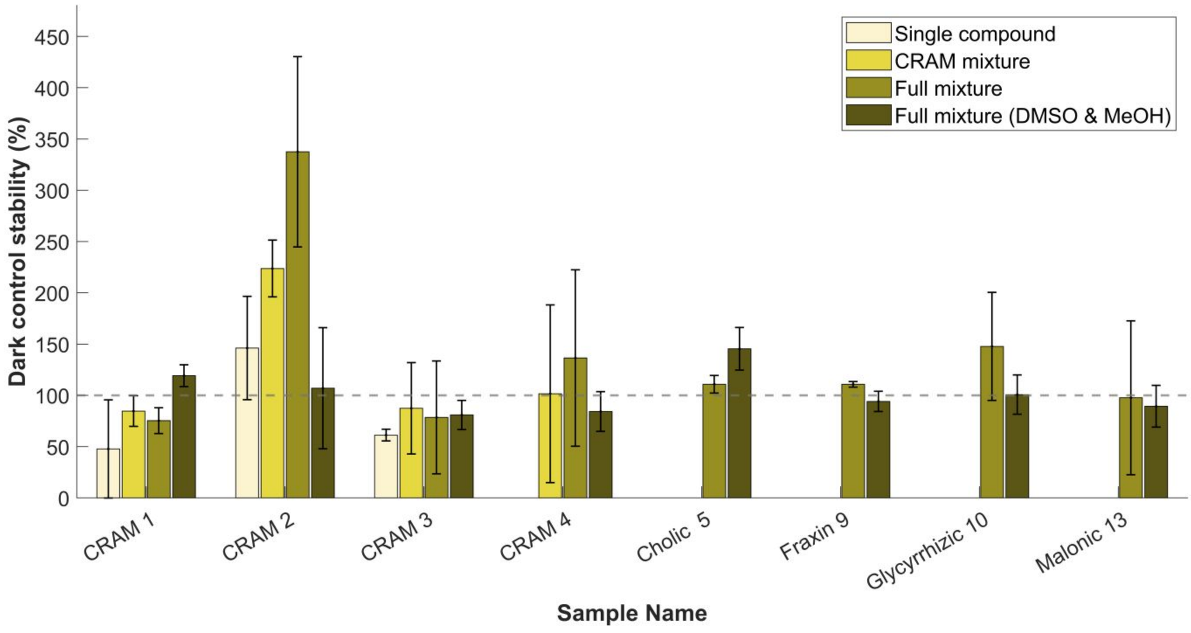

Figure SI6: *Dark control vs t0 Full mixture in the respective solvent (MQ or MQ with 1M DMSO and MeOH) for 93 hr experiments. These tests were to check for loss of compound due to sorption, precipitation, hydrolysis etc. 'CRAMmix' refers to the mixture of compounds* ***1****-****4****. 'Megamix' refers to the full mixture* ***1****-****13****. Note that the 'Single compound' has an expected value of ~50%, because only one isomer (e.g. CRAM* ***1a****) was tested, and the t0 Full mix had both isomers present. The CRAM mixture and full mixture bars do not have this issue, and should be 100% for full recovery.*

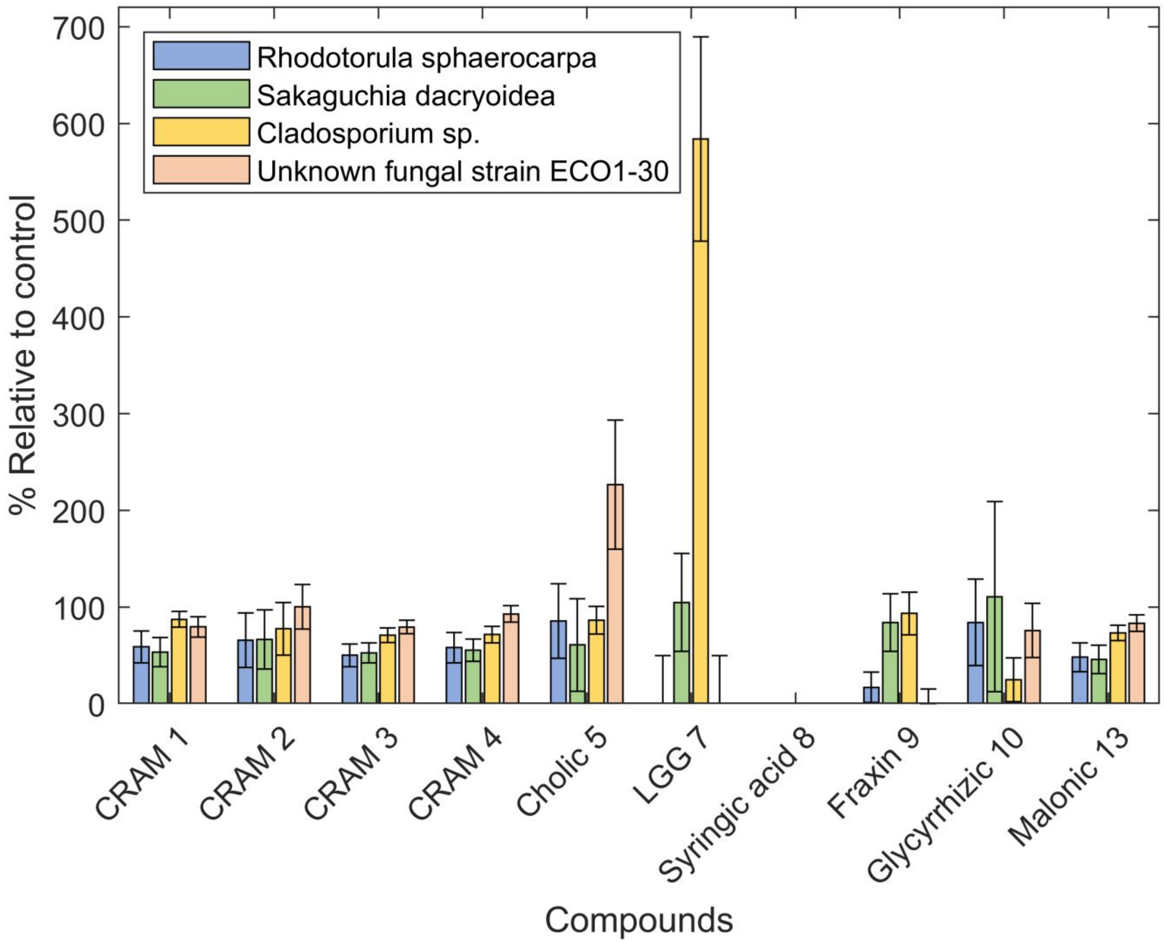

Figure SI7: *Percent remaining values for 10 compound groups detected by UPLC-ESI-HRMS after solid phase extraction for the 102-day biological incubations in artificial seawater with selected strains of marine fungi. *Syringic acid not detected in tests or controls. The very high values found for LGG (Cladosporium sp.) and Cholic acid (Unknown fungal strain ECO1-30) could signify features with these masses and retention times were produced during these incubations, or that the compounds degraded in the control samples. Either way, we do not have evidence that these compounds were removed in the experiments.*

<u>Experimental Considerations</u>

The analytical experimental values shown in Figures 2 and 3 have only moderate precision and accuracy due to various factors. These include 1) biological variability and general bottle effects, 2) the summing of several isomers for a single reported value, 3) lack of accurate internal standards for quantification, because hippuric acid was used for all compounds, and it elutes separately and as such cannot account for all variability in ionization suppression, 4) the fact that experiments were done on complex mixtures of spiked compounds and not individually, meaning one compound (e.g. CRAM 311)

could in principle be modified into another (e.g. CRAM267), 5) the low concentrations used (10 ppb), which was to keep conditions relatively realistic. For these reasons, we focus our discussion on whether individual compounds largely remained (>50% left), remained only in trace quantities, or were essentially removed.

UV-Visible Spectra Aqcuisition

UV-visible spectra were acquired on an Ultimate 3000 (Thermo Fischer) with an ISQ EC single quadrupole MS, using a Phenomenex Kinetix C18 column (50 x 3 mm, 2.6 µm), with a gradient from 5-100% acetonitrile in water with 0.05% formic acid, and samples being injected at 2 uL volumes from approximately 100 ppm samples. LCMS reports including TIC data followed by UV-data at 214, 254, and 280 nm are included. Where peaks are absent in the set UV-wavelengths, TIC data is used to identify the presence of specific compounds or impurities. Data is listed for individual compounds, with this LCMS report, followed by UV-visible absorbance spectra from 200-350 nm for any detected peak (including their retention time), followed by UV-visible absorbance spectra from 200-350 nm for the entirety of the LCMS run excluding the void (from around 0.25 minutes to the end of the LCMS run at 3 minutes). These UV-spectra integrated across the entire LC run are used to check for any potential impurities that absorb strongly at wavelengths not included in the LCMS reports. It is worth noting that this LCMS system is not the same as that used for quantification in the general experiments, and resolution of diastereomers for the CRAM compounds is much worse. Also important to note is that the scale of absorbance varies between compounds, and while minor impurities in compounds such as CRAM311 **4** (Figure SI23) show absorption at ~300 nm, these are below 1 mAU, while the absorbance in this range for a compound like fraxin **9** is around 100 mAU (Figure SI37).

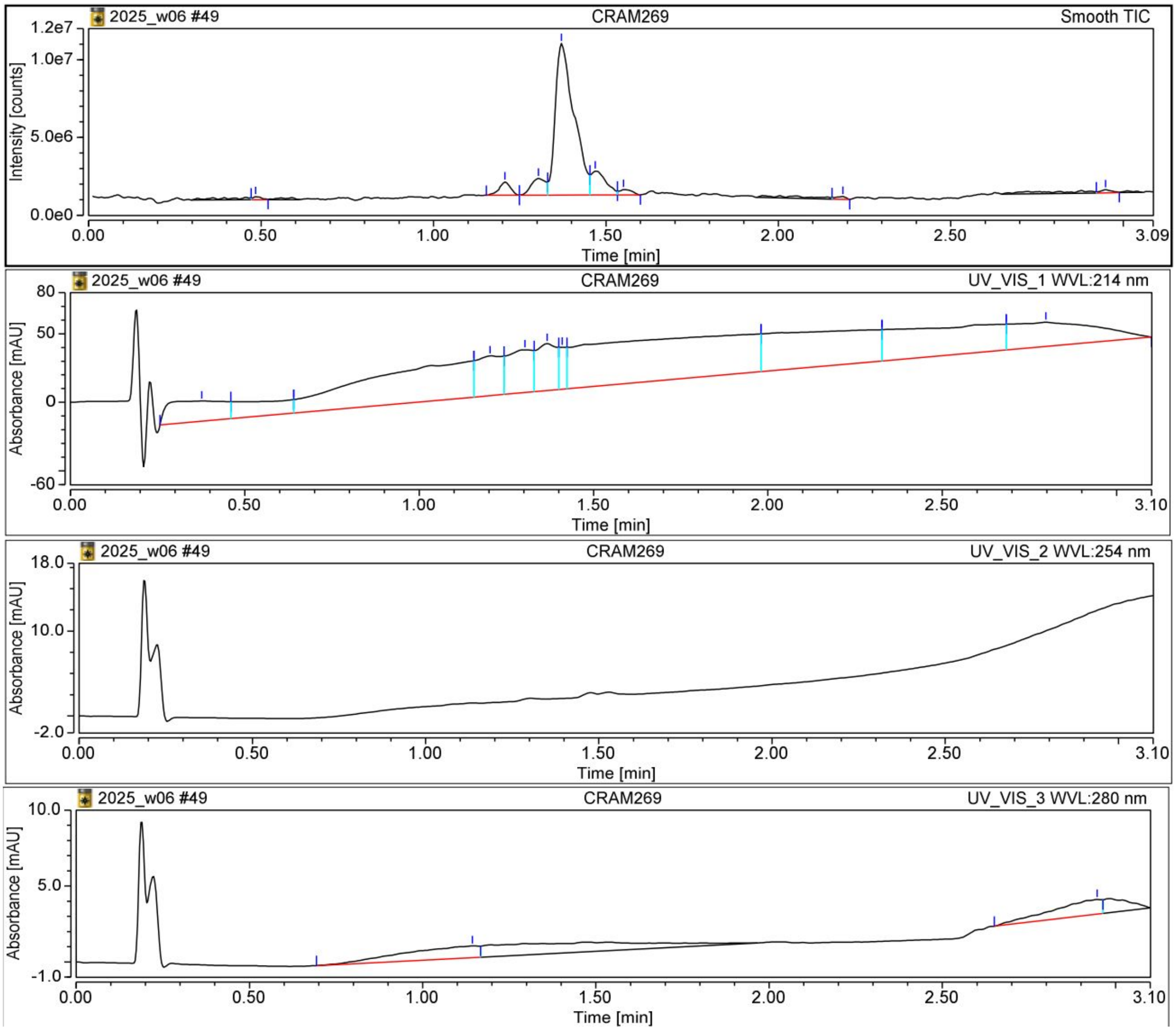

Figure SI8*: LCMS report for CRAM* ***1****.*

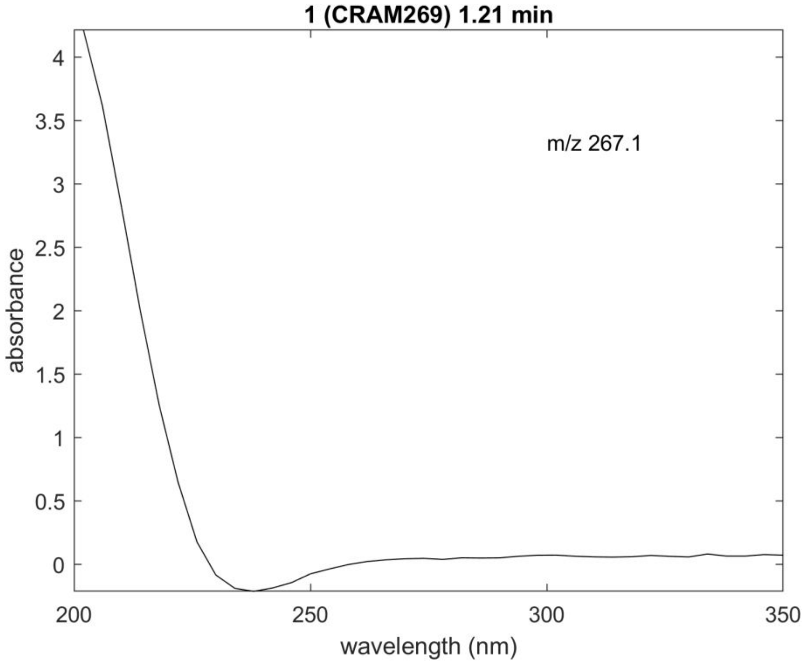

Figure SI9: *UV-visible spectrum for CRAM* ***1*** *retention time 1.21 minutes.*

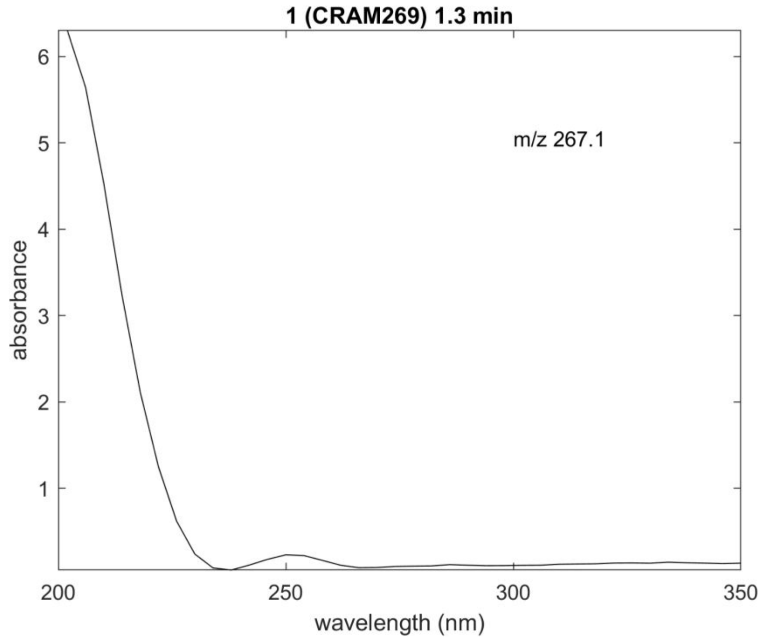

Figure SI10: *UV-visible spectrum for CRAM **1** retention time 1.30 minutes.*

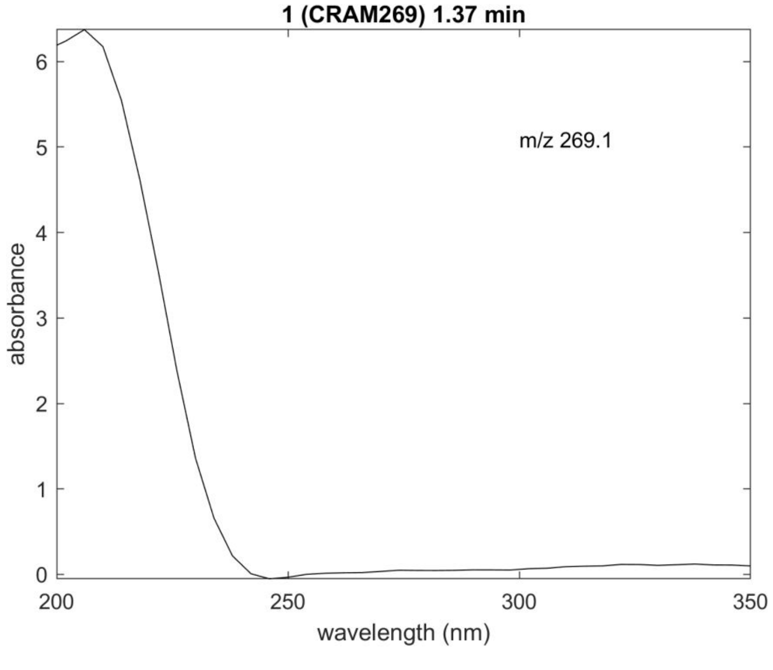

Figure SI11: *UV-visible spectrum for CRAM **1** retention time 1.37 minutes.*

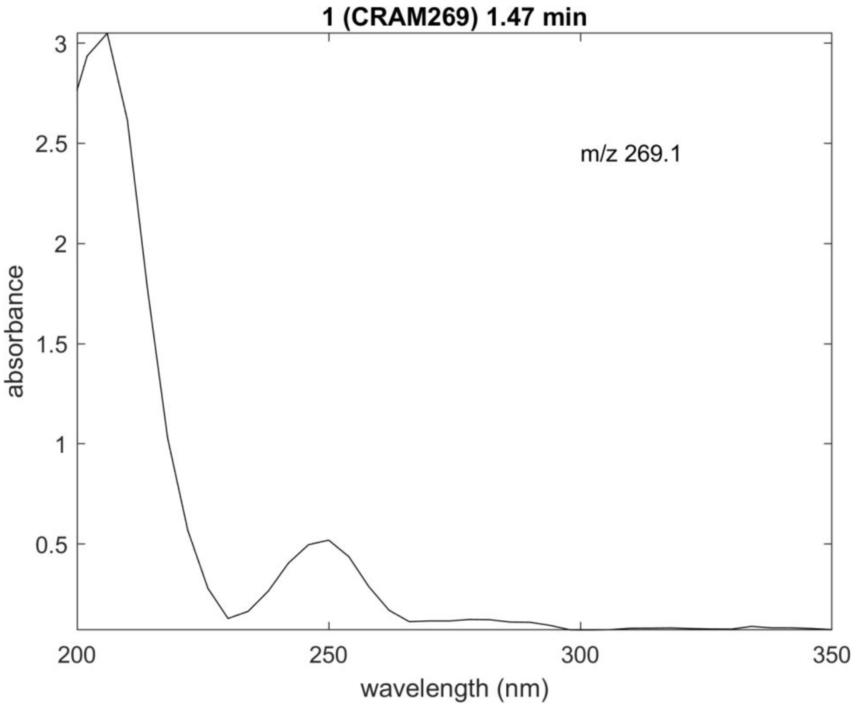

Figure SI12: *UV-visible spectrum for CRAM* ***1*** *retention time 1.47 minutes.*

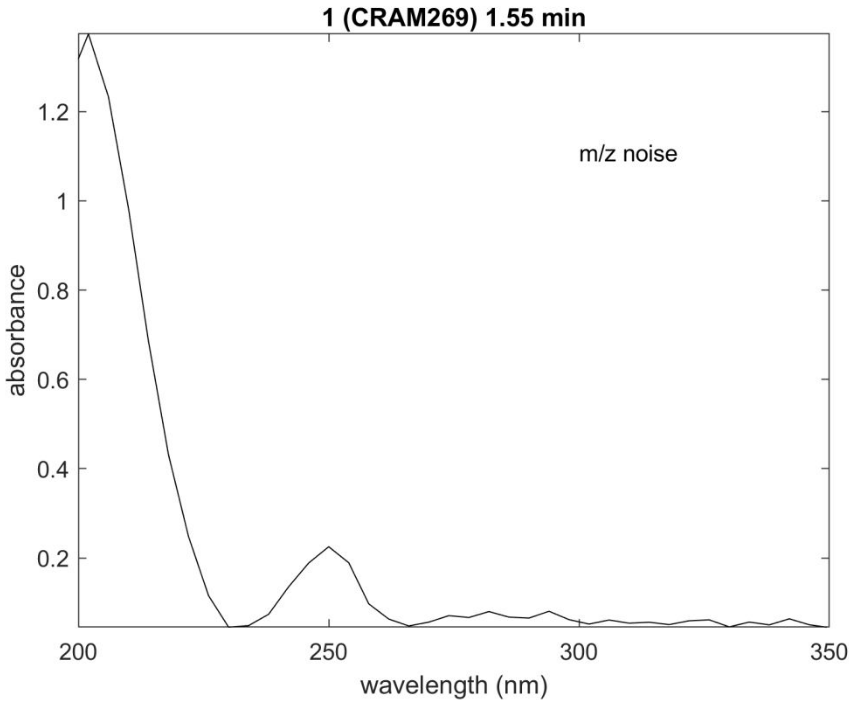

Figure SI13: *UV-visible spectrum for CRAM* ***1*** *retention time 1.55 minutes.*

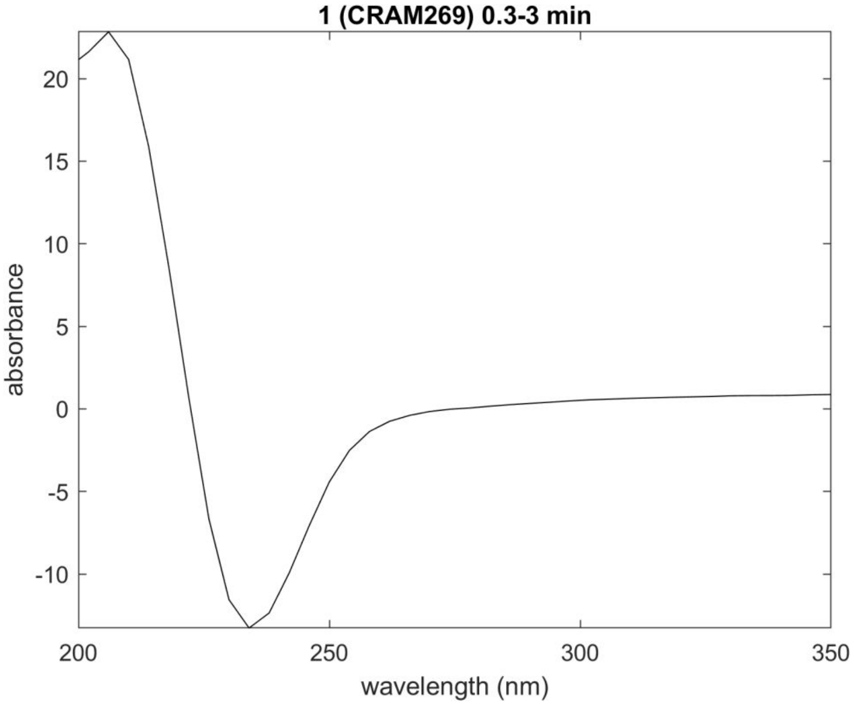

Figure SI14: *UV-visible spectrum for total LC chromatogram of CRAM* ***1****.*

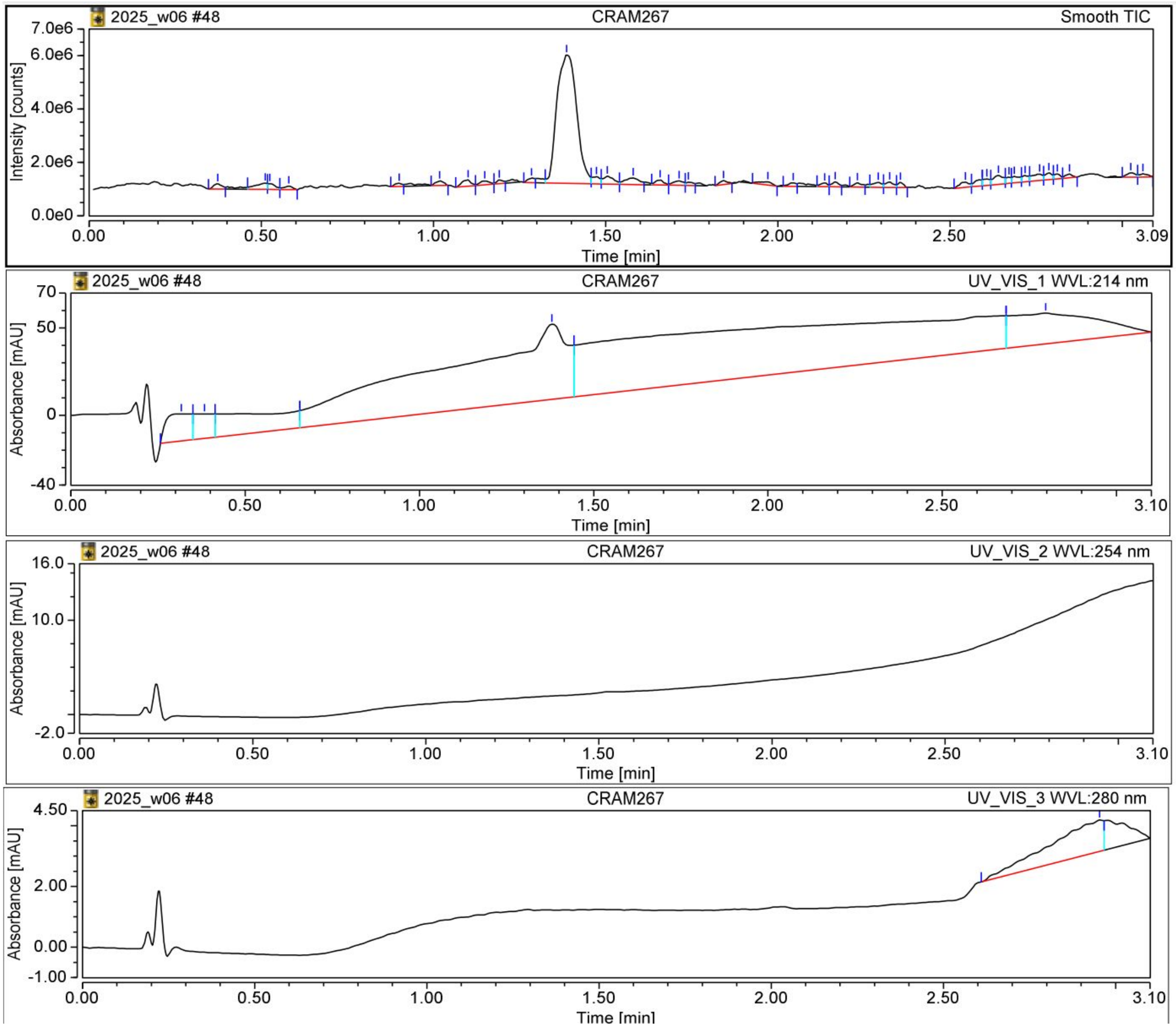

Figure SI15: *LCMS report for CRAM* ***2****.*

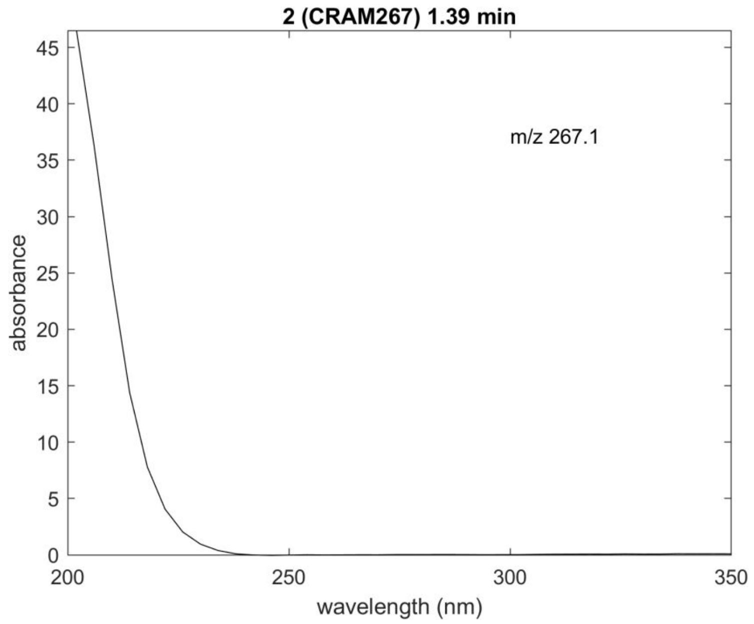

Figure SI16: *UV-visible spectrum for CRAM* ***2*** *retention time 1.39 minutes.*

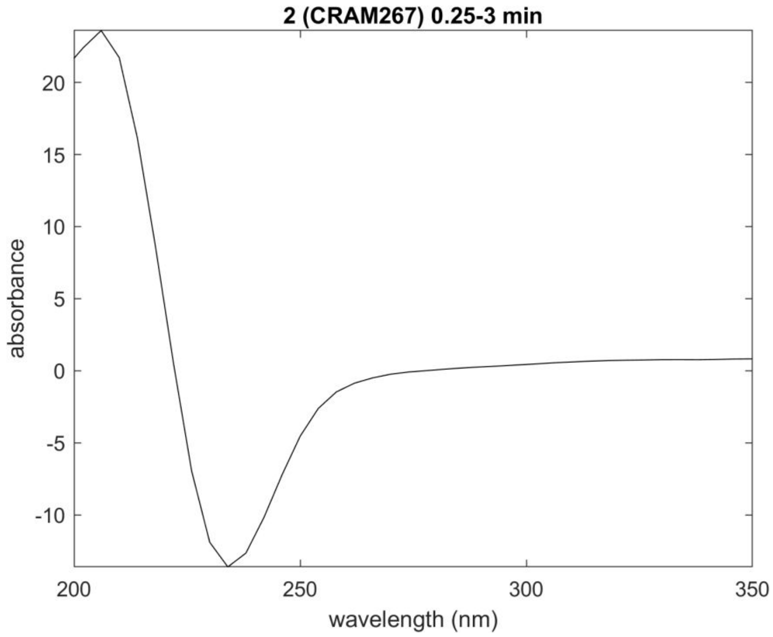

Figure SI17: *UV-visible spectrum for total LC chromatogram of CRAM* ***2****.*

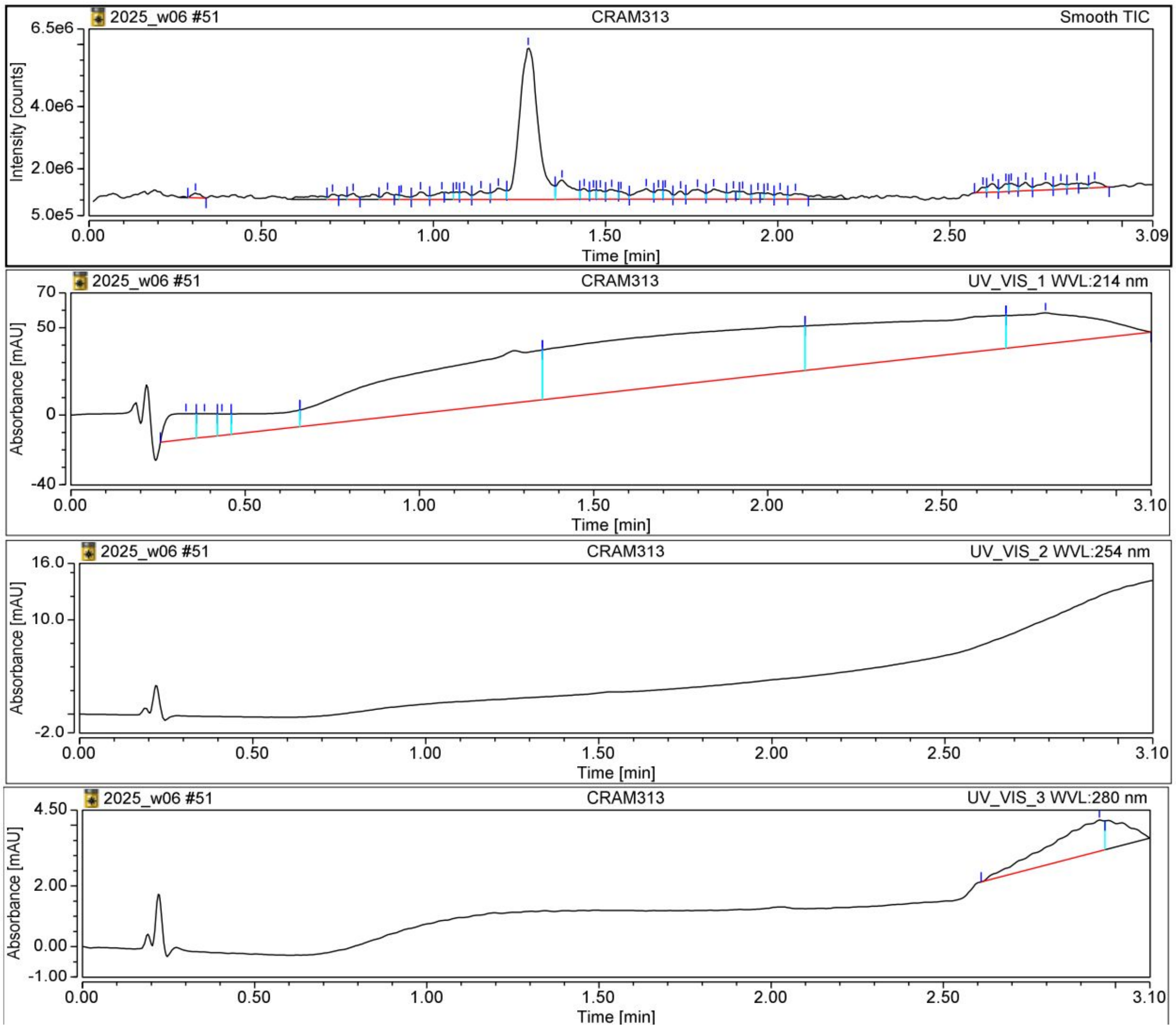

***Figure SI18**: LCMS report for CRAM **3**.*

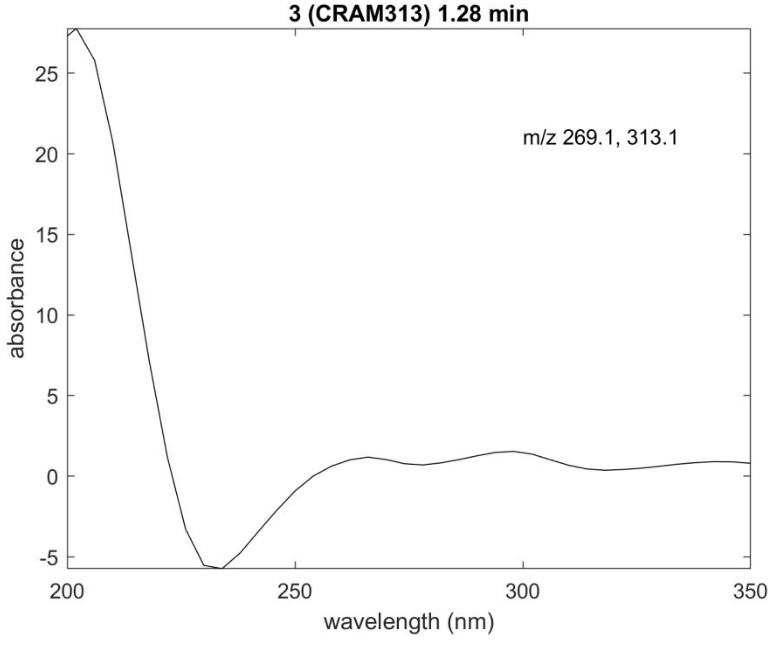

***Figure SI19:** UV-visible spectrum for CRAM **3** retention time 1.28 minutes.*

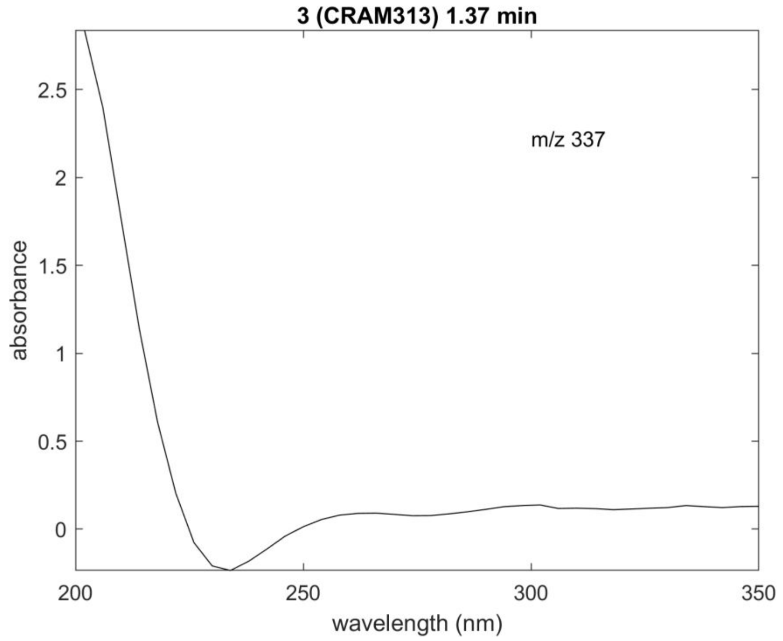

Figure SI20: *UV-visible spectrum for CRAM **3** retention time 1.37 minutes.*

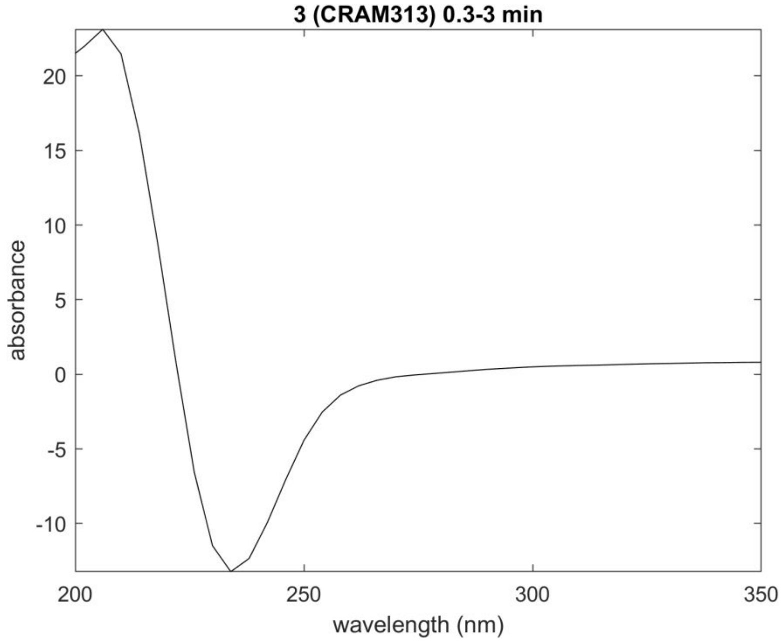

Figure SI21: *UV-visible spectrum for total LC chromatogram of CRAM **3**.*

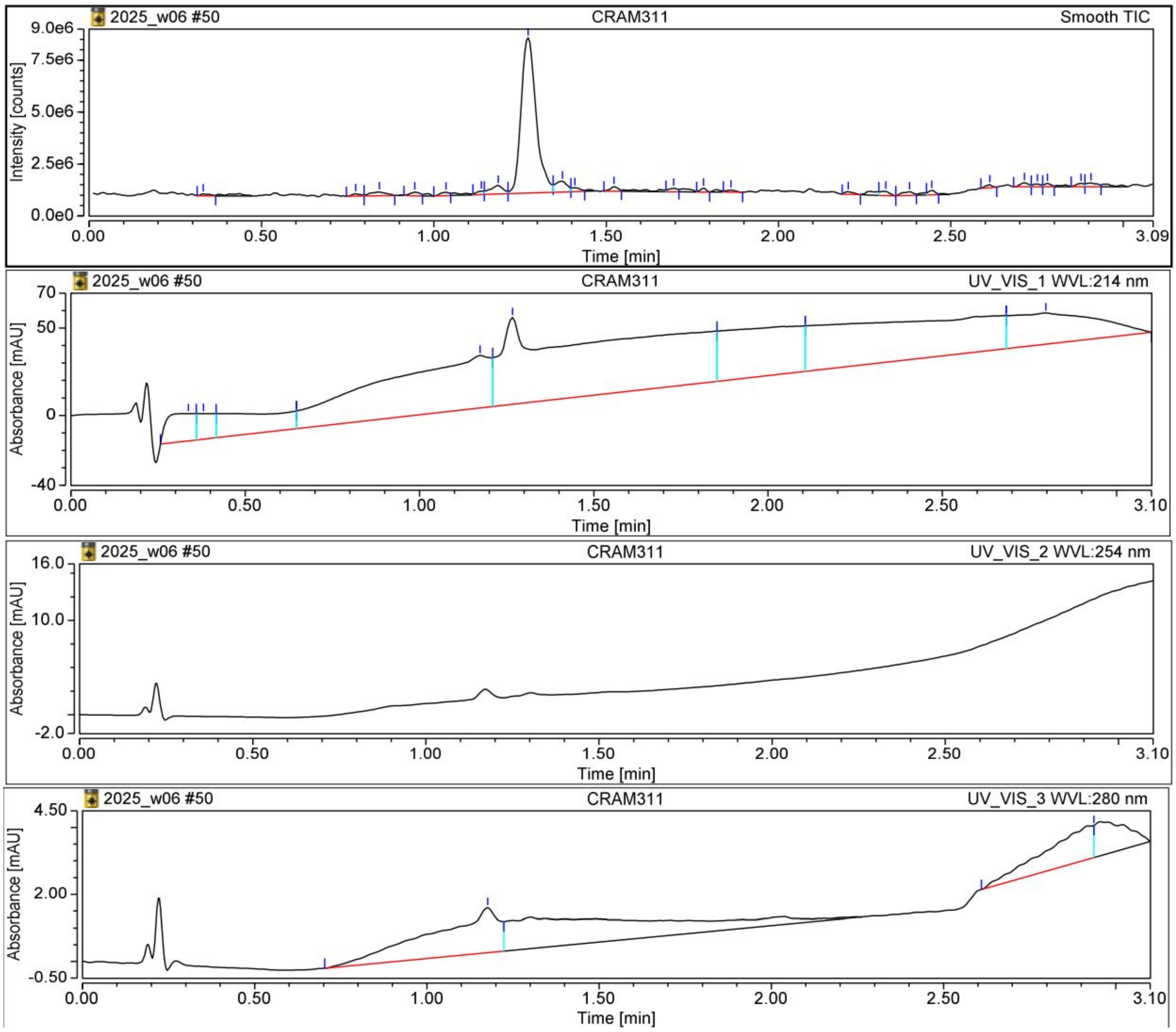

***Figure SI22**: LCMS report for CRAM **4**.*

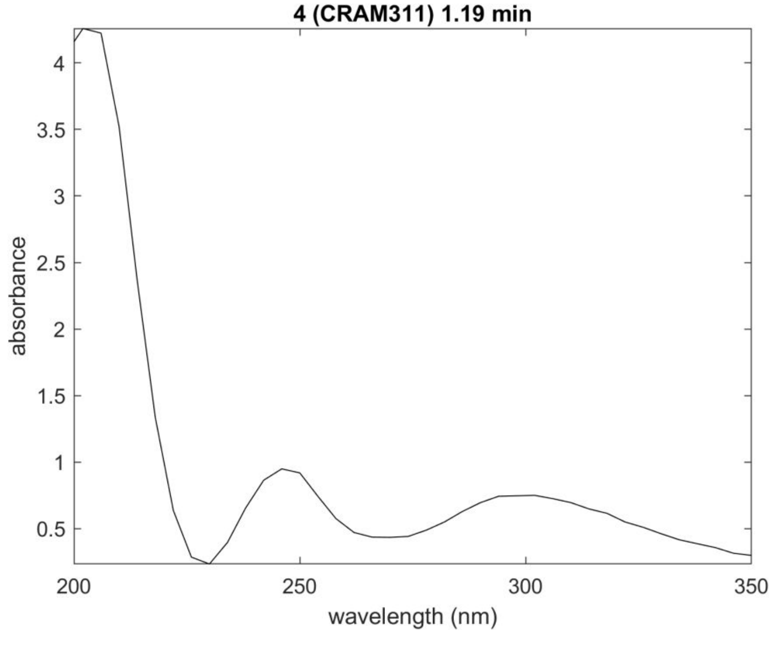

***Figure SI23:** UV-visible spectrum for CRAM **4** retention time 1.19 minutes.*

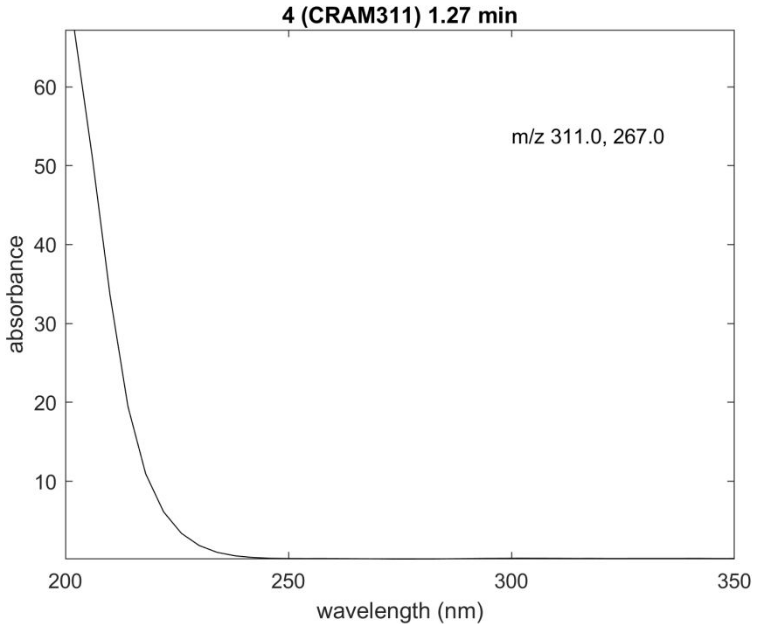

Figure SI24: *UV-visible spectrum for CRAM **4** retention time 1.27 minutes.*

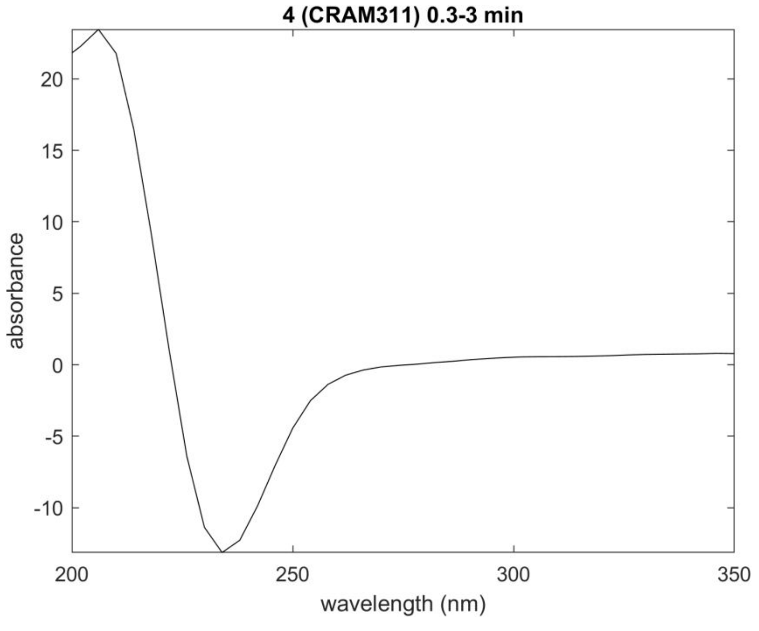

Figure SI25: *UV-visible spectrum for total LC chromatogram of CRAM **4**.*

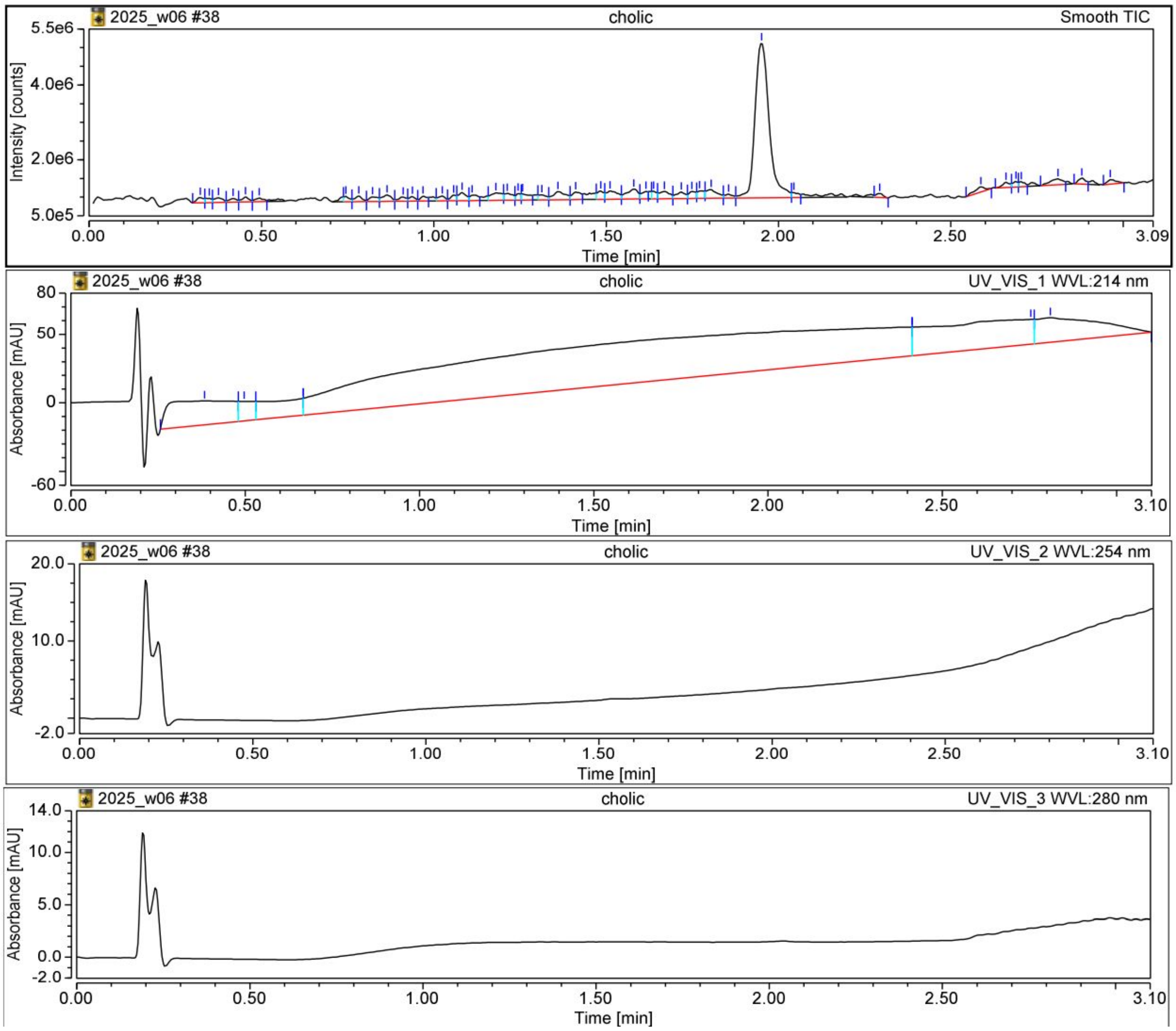

Figure SI26: *LCMS report for cholic acid (**5**).*

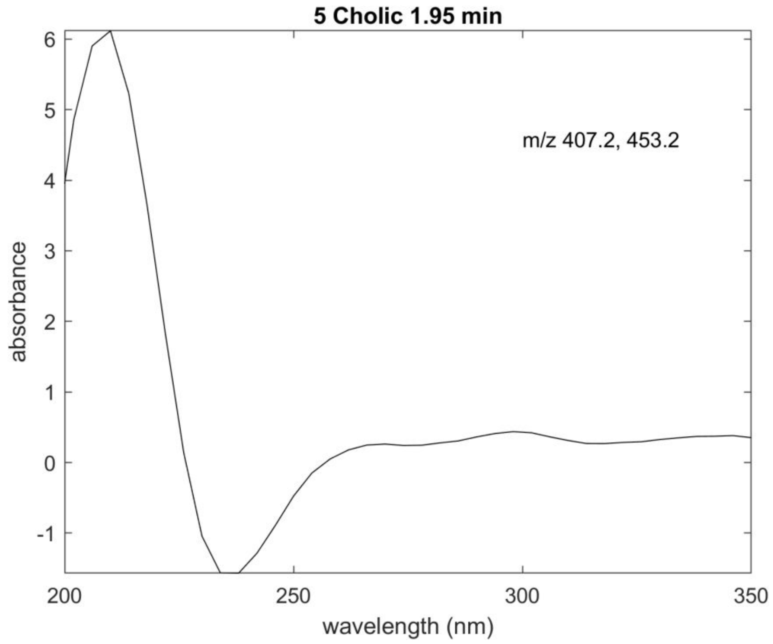

Figure SI27: *UV-visible spectrum for cholic acid (**5**) retention time 1.95 minutes.*

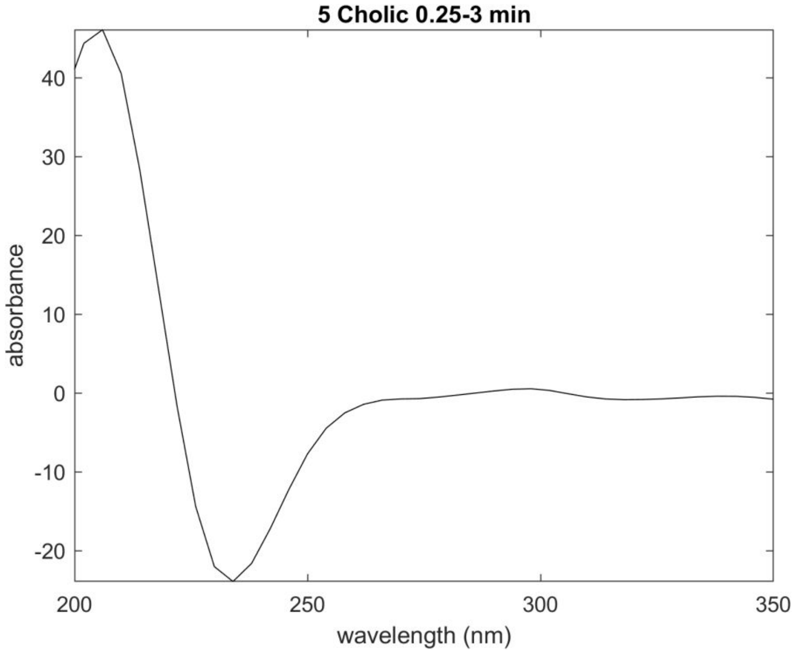

Figure SI28: *UV-visible spectrum for total LC chromatogram of cholic acid (**5**).*

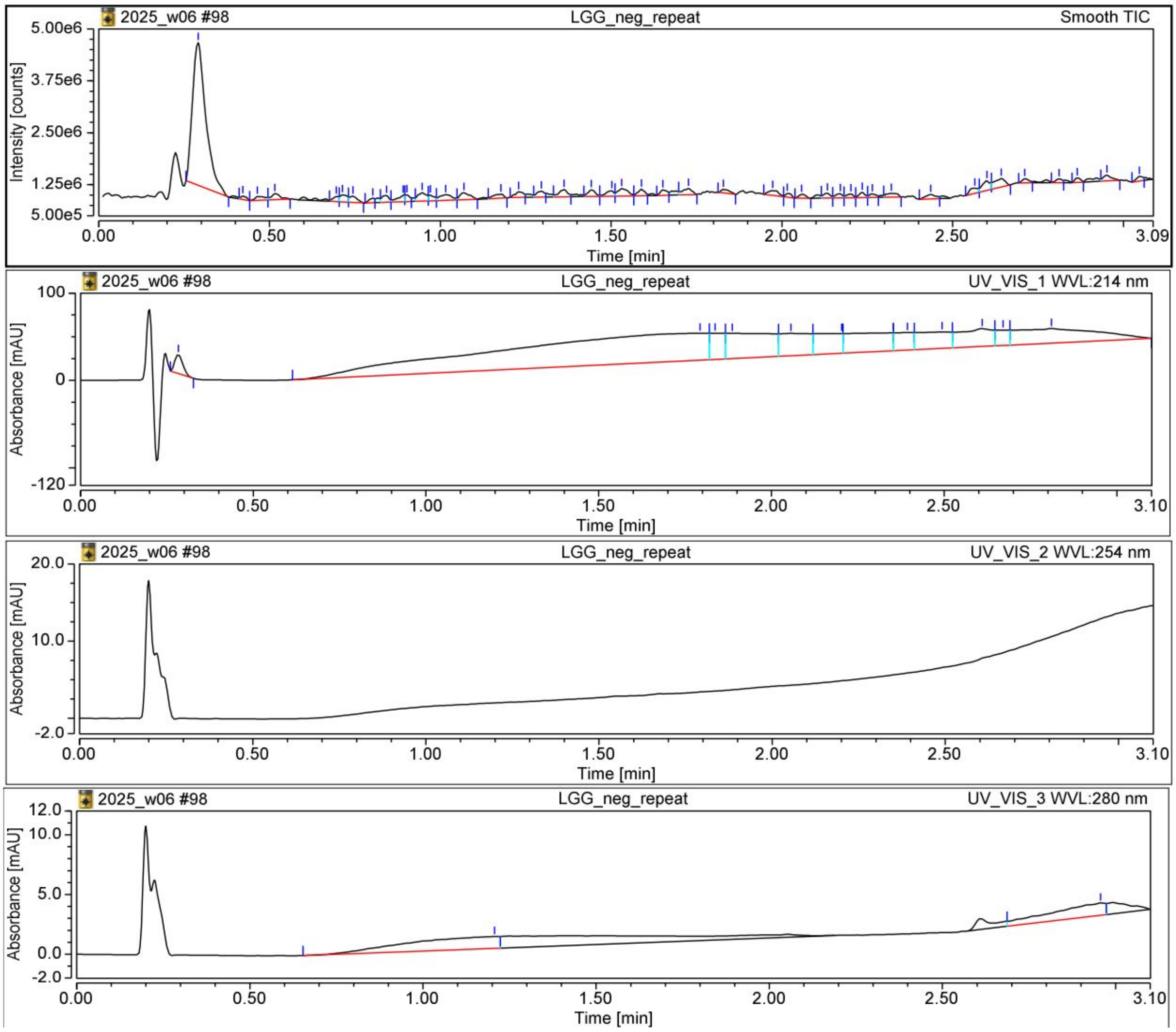

Figure SI29: *LCMS report for LGG* ***7***.

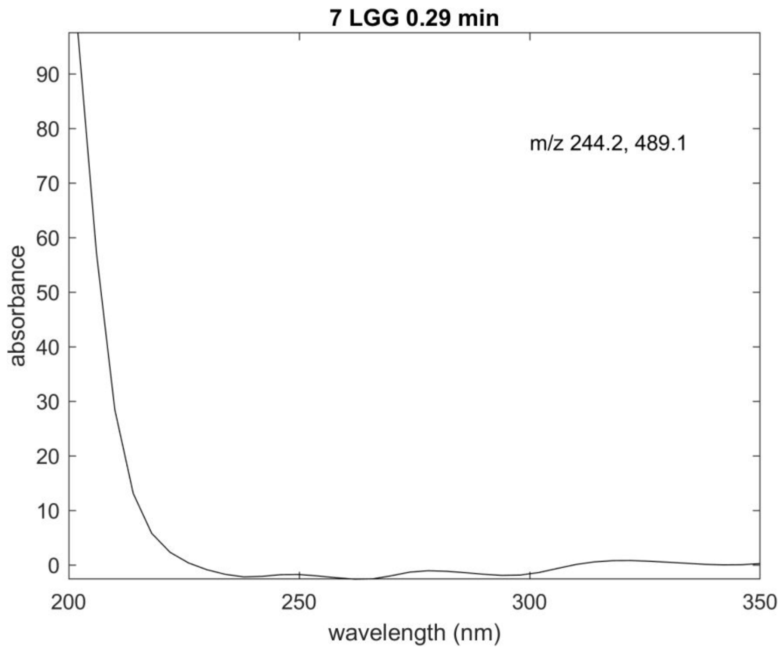

Figure SI30: *UV-visible spectrum for LGG* ***7*** *retention time 0.29 minutes.*

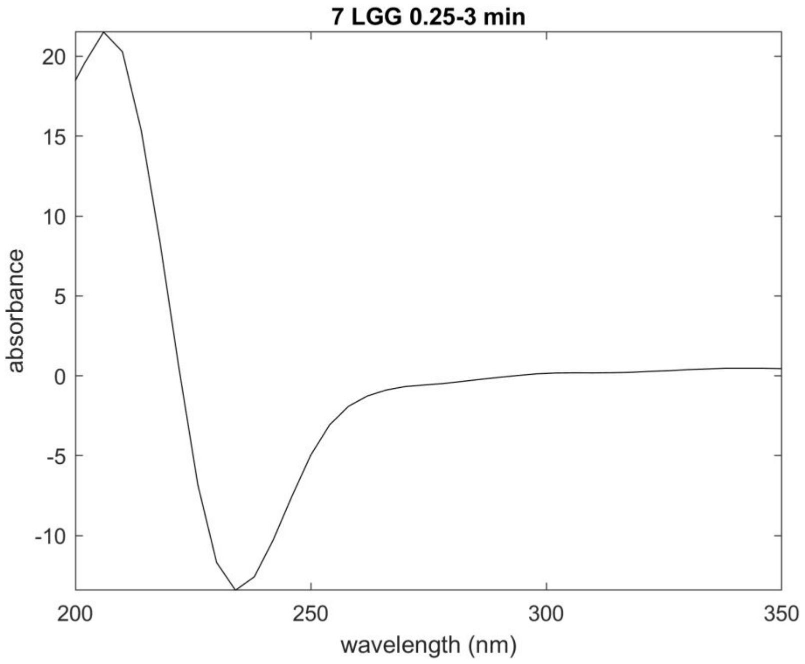

Figure SI31: *UV-visible spectrum for total LC chromatogram of LGG **7**.*

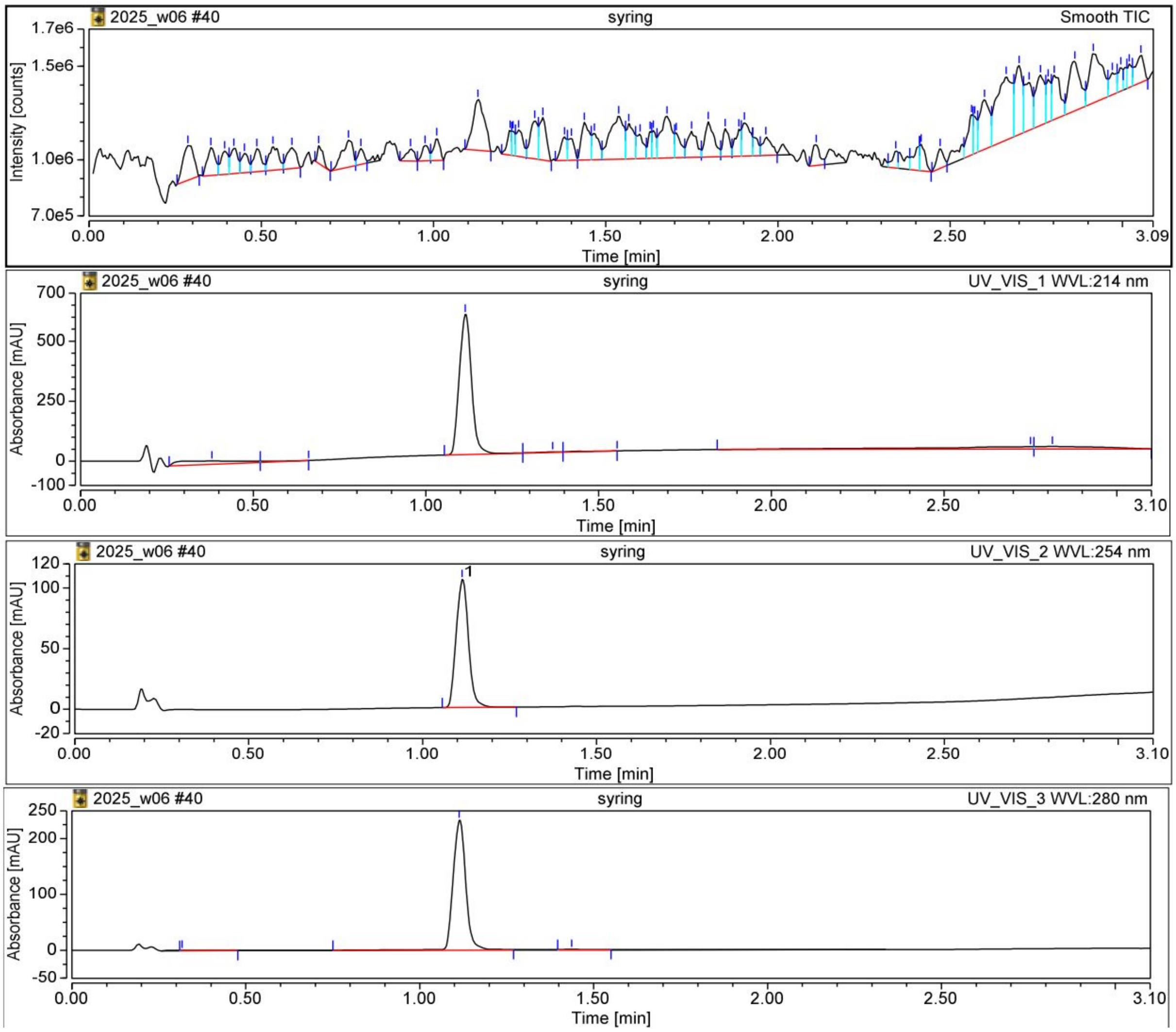

***Figure SI32**: LCMS report for syringic acid **8**.*

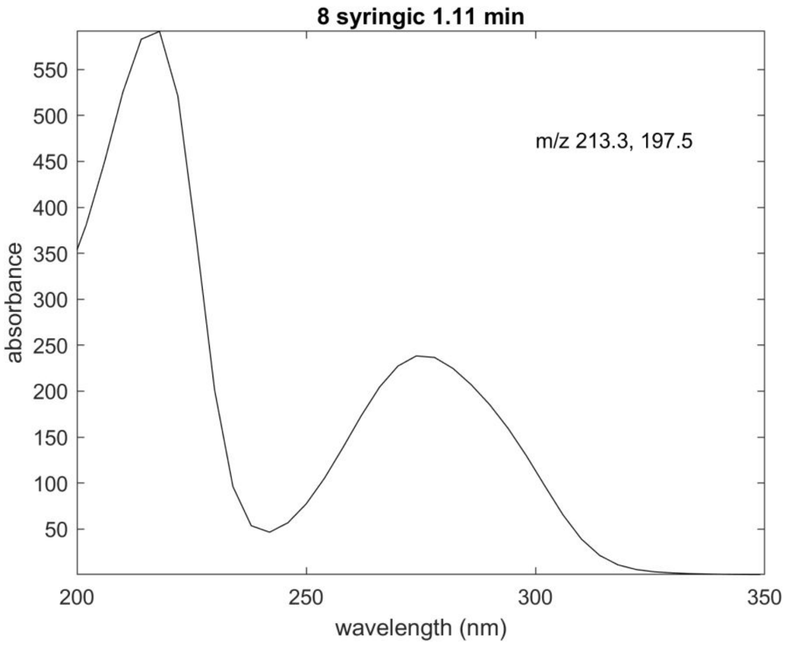

***Figure SI33:** UV-visible spectrum for syringic acid **8** retention time 1.11 minutes.*

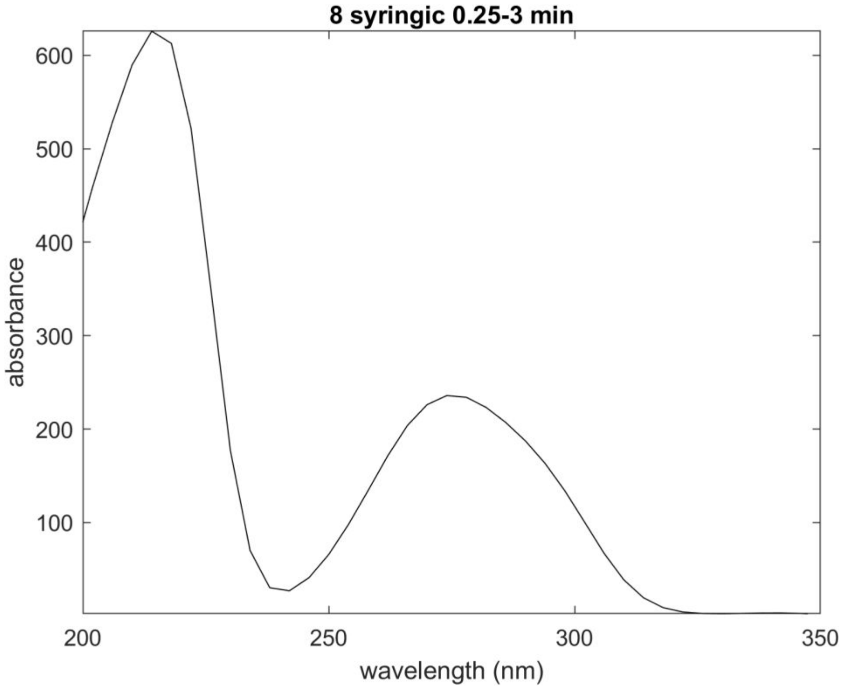

Figure SI34: *UV-visible spectrum for total LC chromatogram of syringic acid* ***8****.*

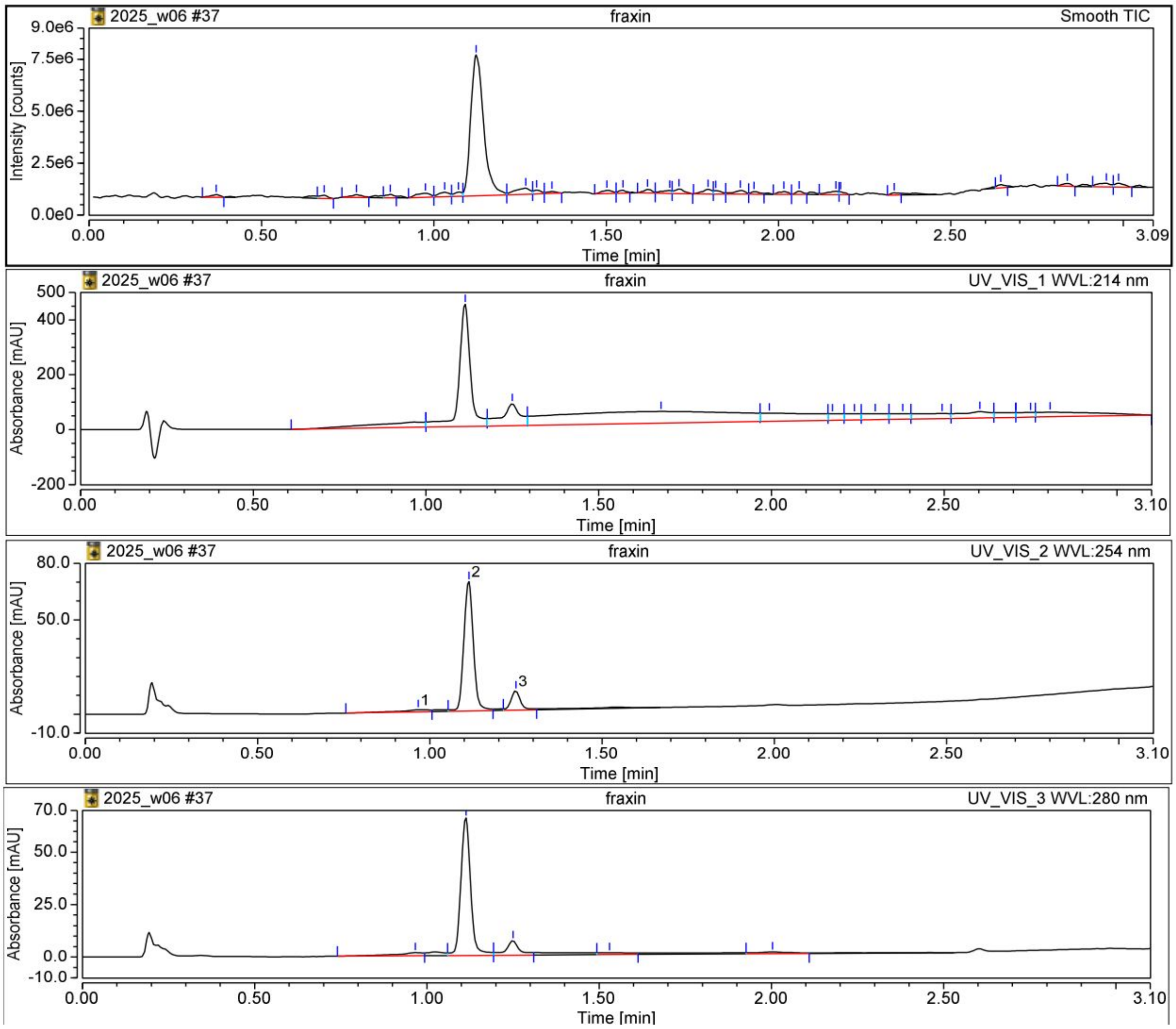

Figure SI35*: LCMS report for fraxin* ***9****.*

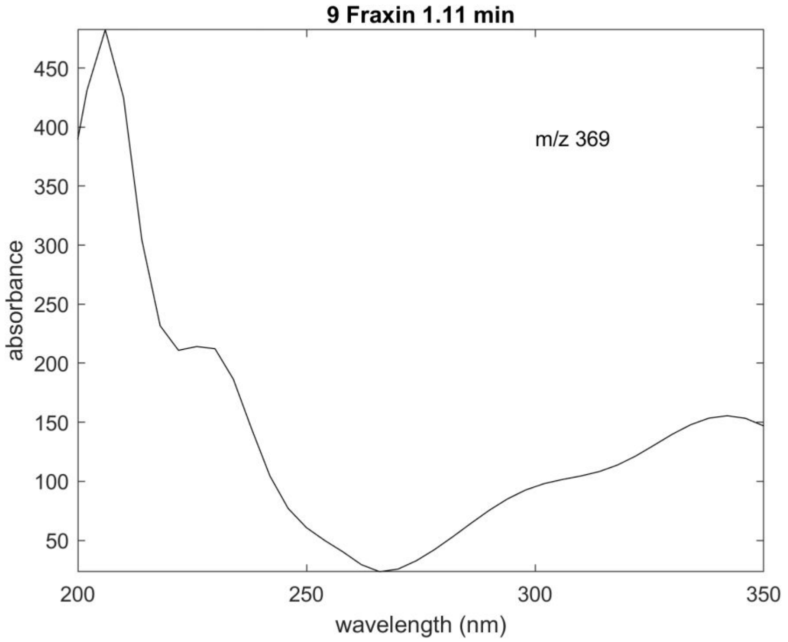

Figure SI36: *UV-visible spectrum for fraxin* ***9*** *retention time 1.11 minutes.*

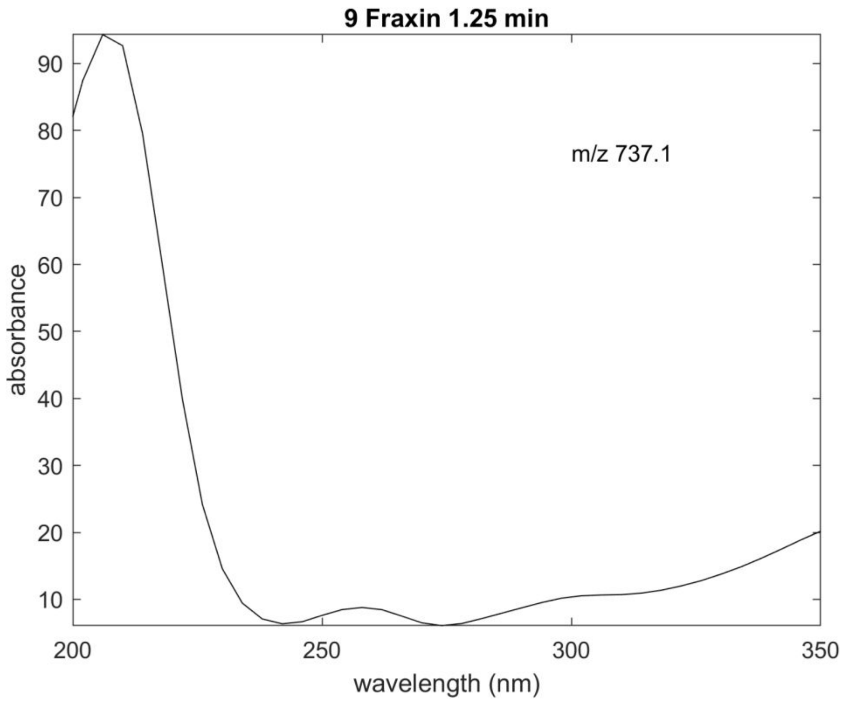

Figure SI37: *UV-visible spectrum for fraxin* ***9*** *retention time 1.25 minutes.*

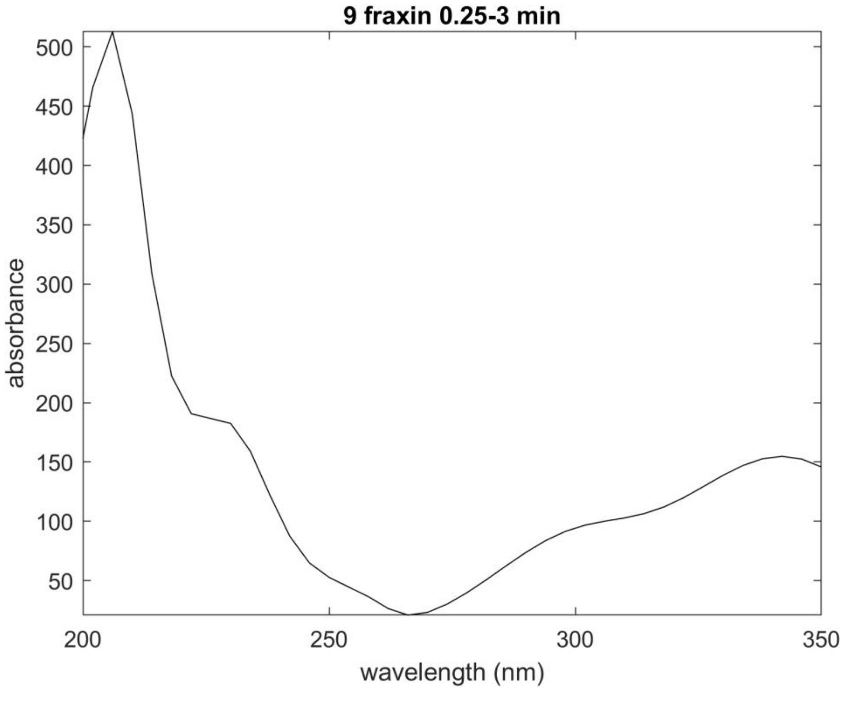

Figure SI38: *UV-visible spectrum for total LC chromatogram of fraxin* ***9****.*

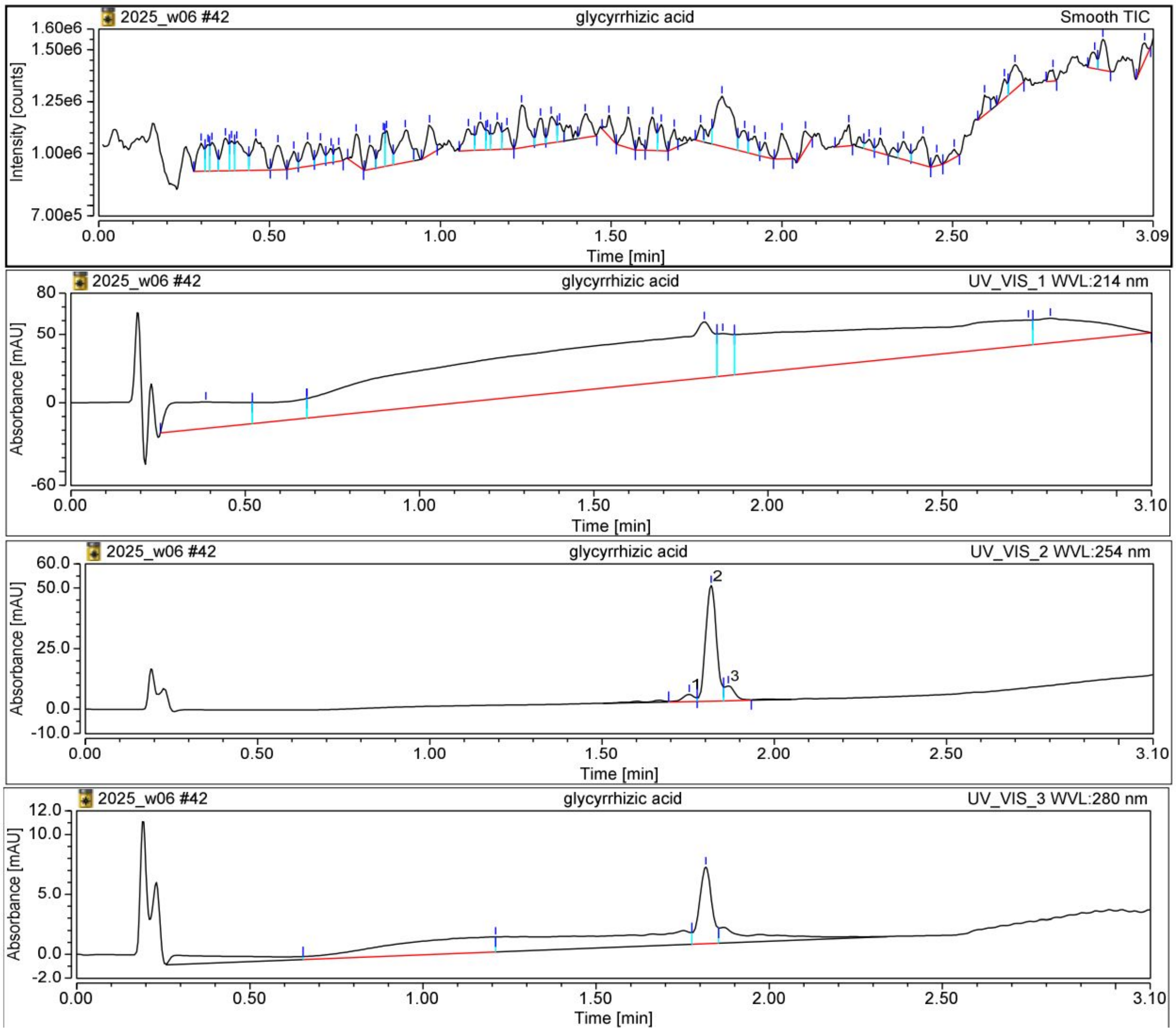

***Figure SI39**: LCMS report for glycyrrhizic acid **10**.*

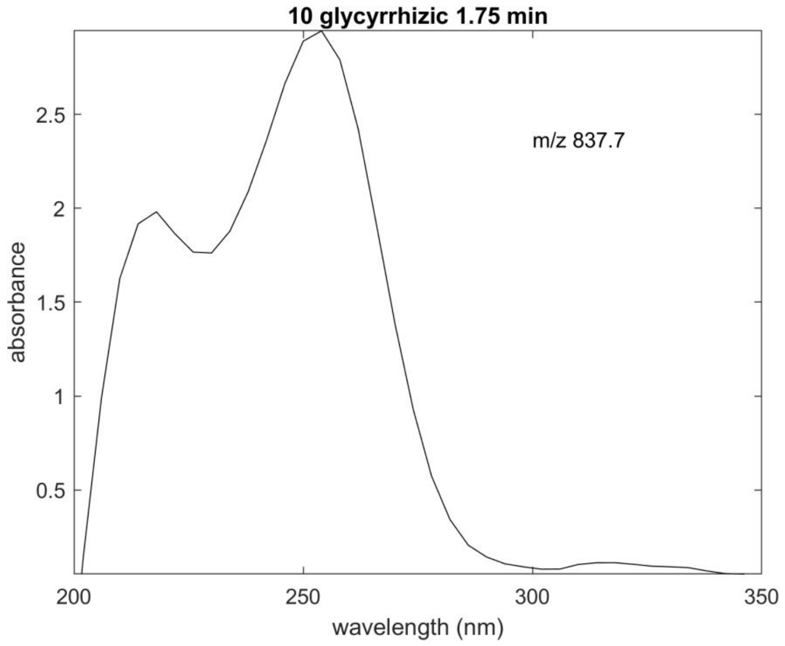

***Figure SI40:** UV-visible spectrum for glycyrrhizic acid **10** retention time 1.75 minutes.*

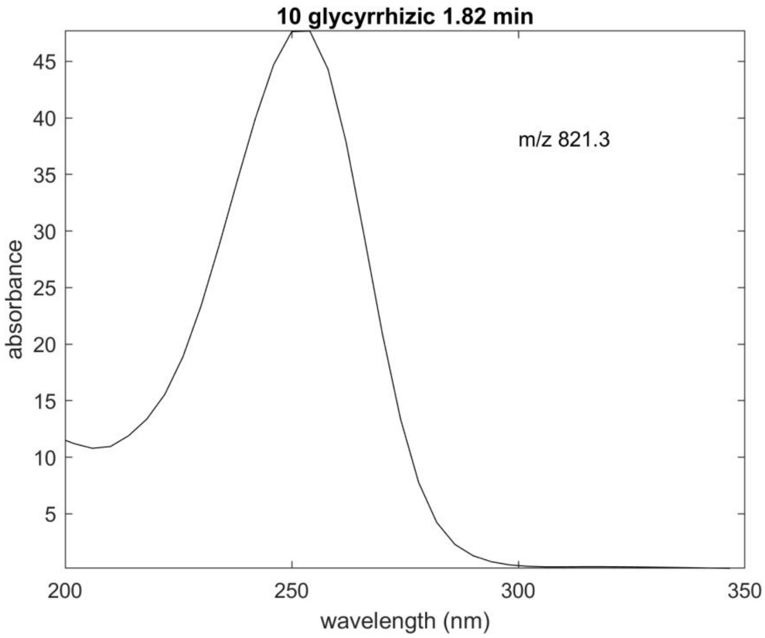

Figure SI41: *UV-visible spectrum for glycyrrhizic acid* ***10*** *retention time 1.82 minutes.*

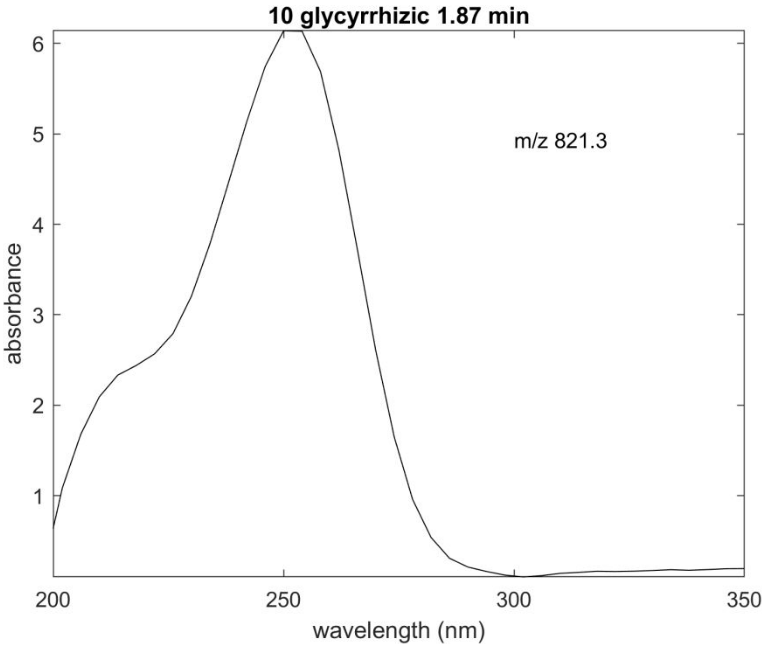

Figure SI42: *UV-visible spectrum for glycyrrhizic acid* ***10*** *retention time 1.87 minutes.*

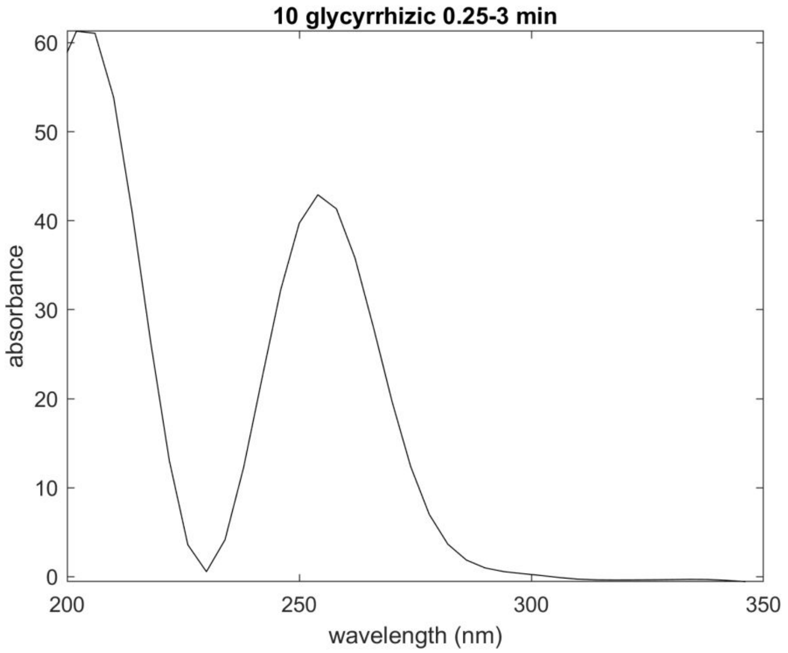

Figure SI43: *UV-visible spectrum for total LC chromatogram of glycyrrhizic acid* ***10****.*

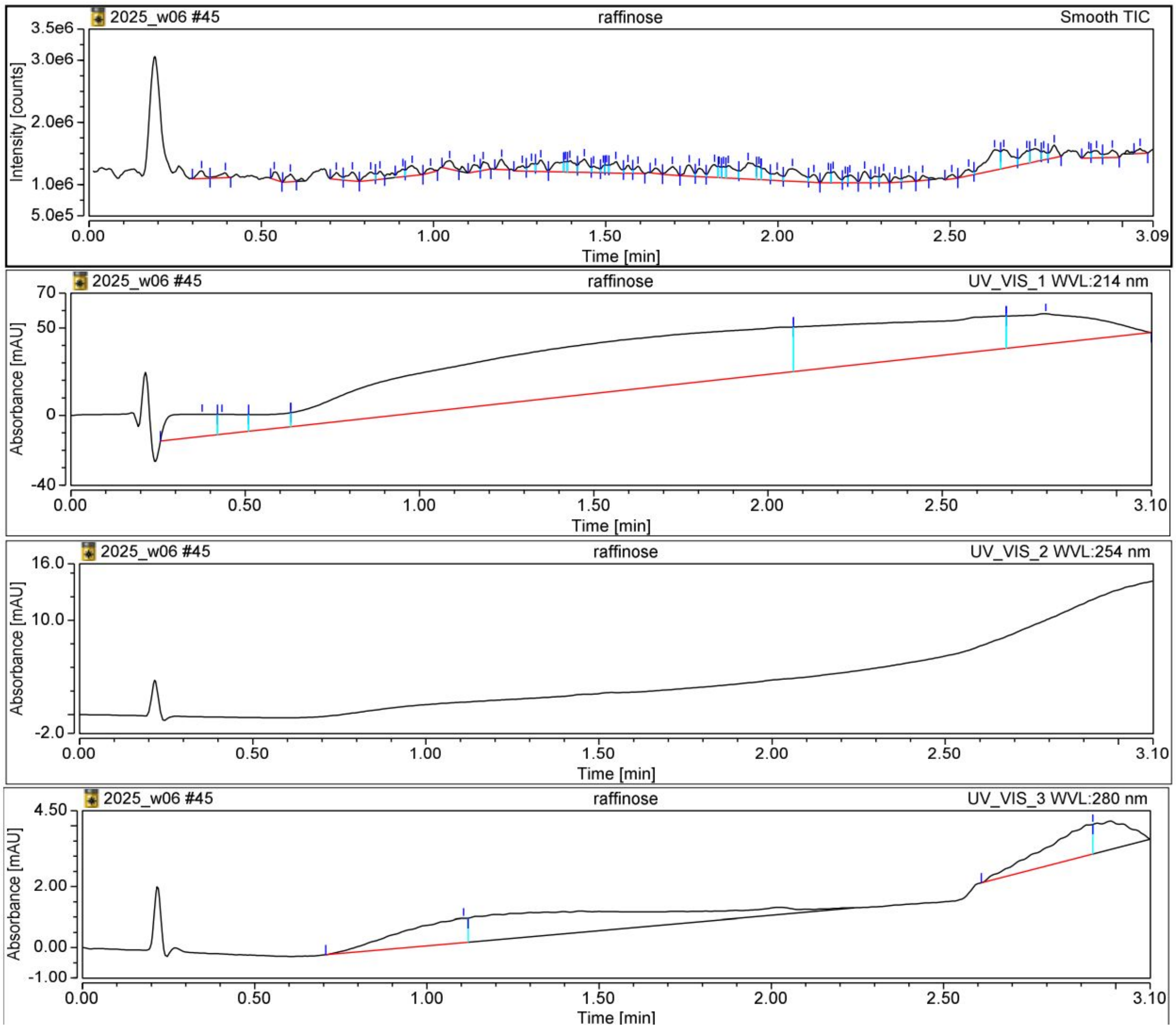

***Figure SI44**: LCMS report for raffinose **11**.*

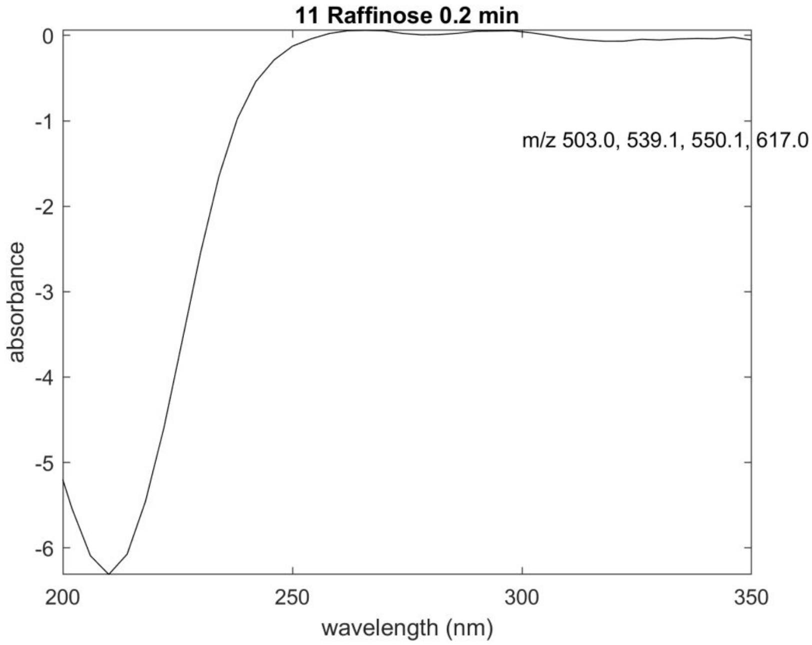

***Figure SI45:** UV-visible spectrum for raffinose **11** retention time 0.20 minutes.*

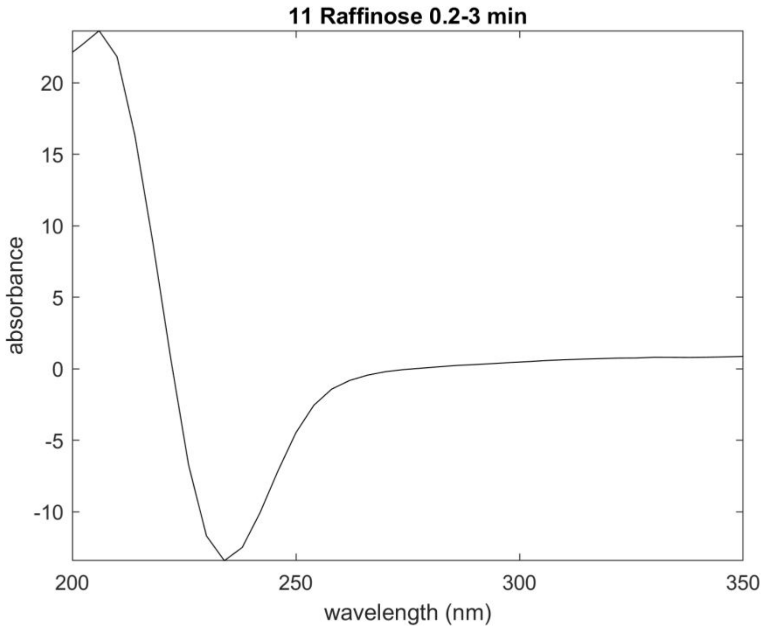

Figure SI46: *UV-visible spectrum for total LC chromatogram of raffinose* ***11****.*

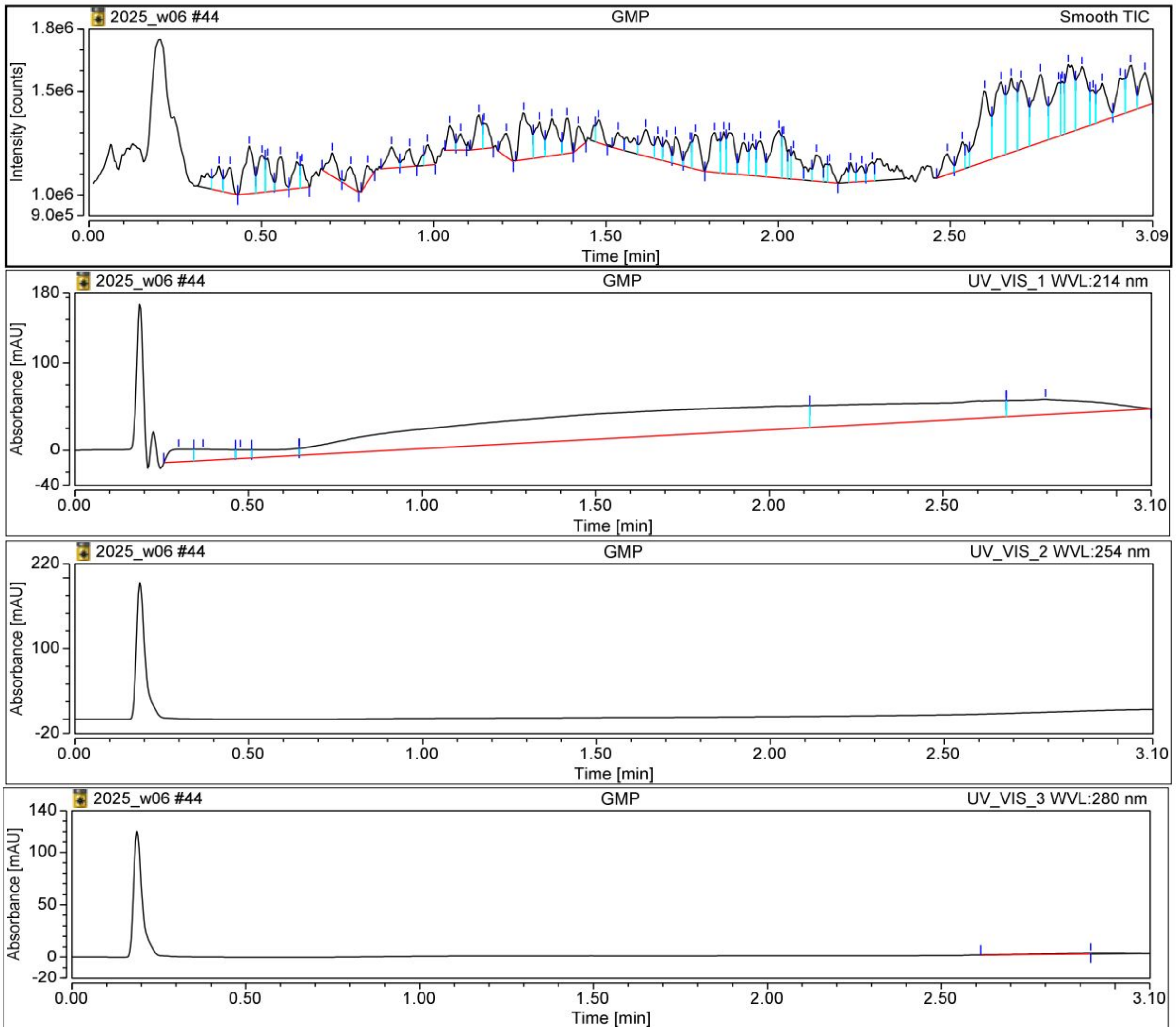

***Figure SI47**: LCMS report for GMP **12**.*

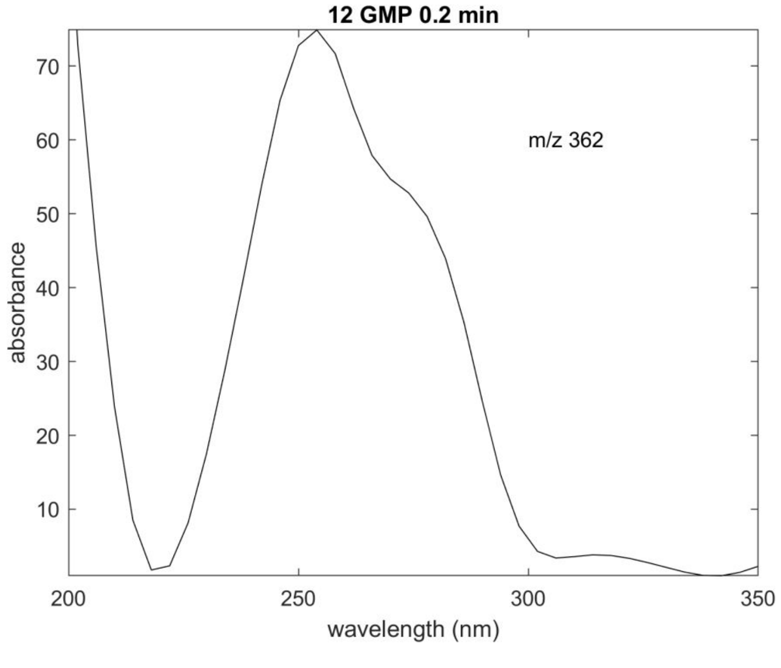

***Figure SI48:** UV-visible spectrum for GMP **12** retention time 0.20 minutes.*

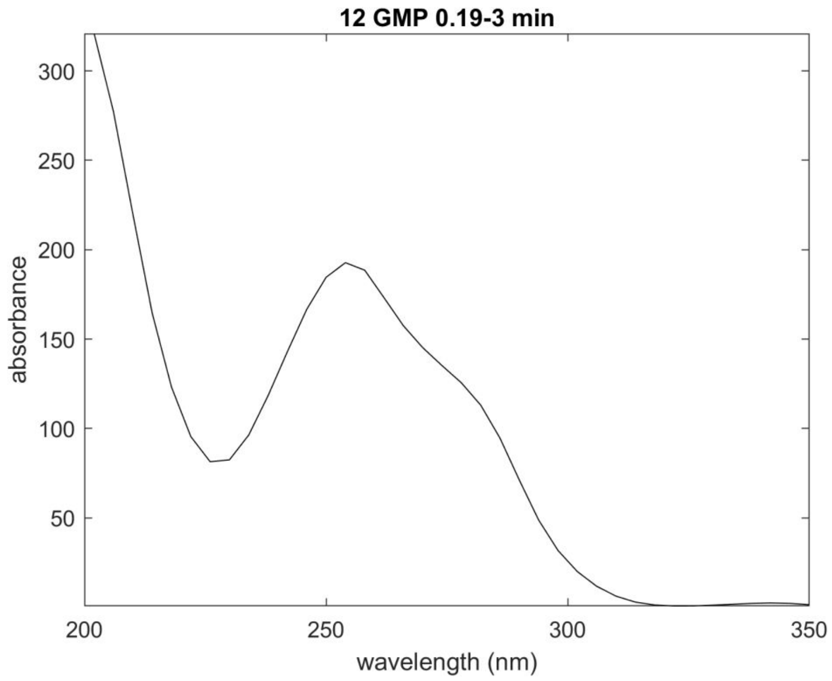

Figure SI49: *UV-visible spectrum for total LC chromatogram of GMP* ***12****.*

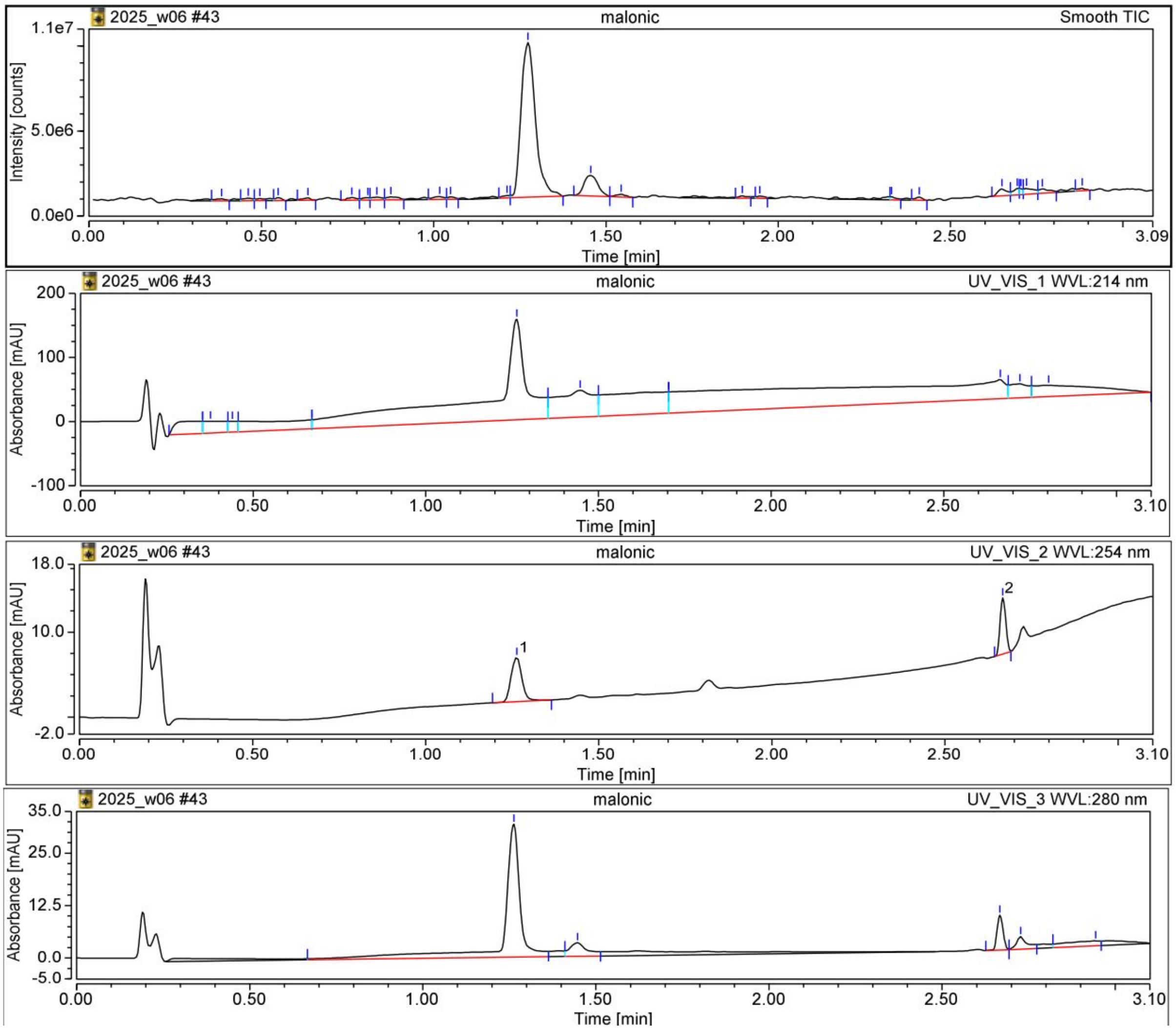

***Figure SI50**: LCMS report for malonic tetraacid **13**.*

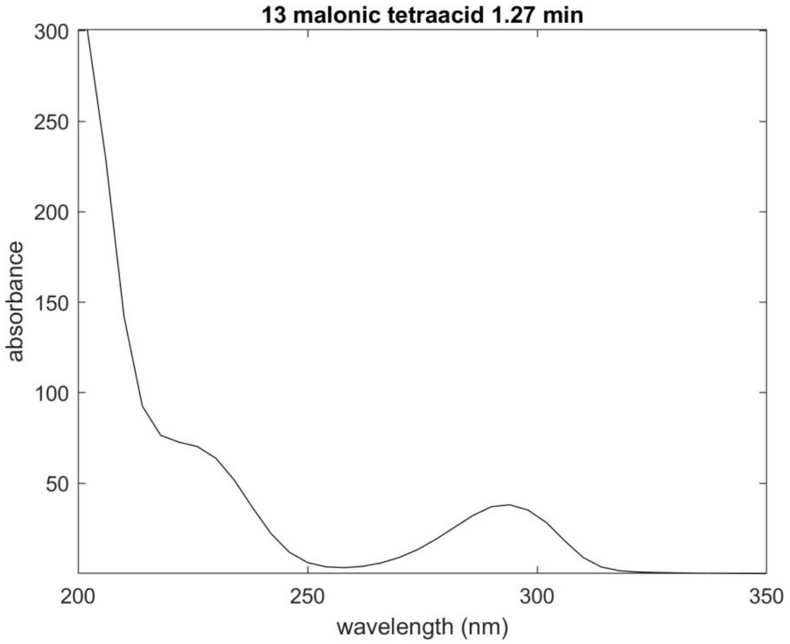

***Figure SI51:** UV-visible spectrum for malonic tetraacid **13** retention time 1.27 minutes.*

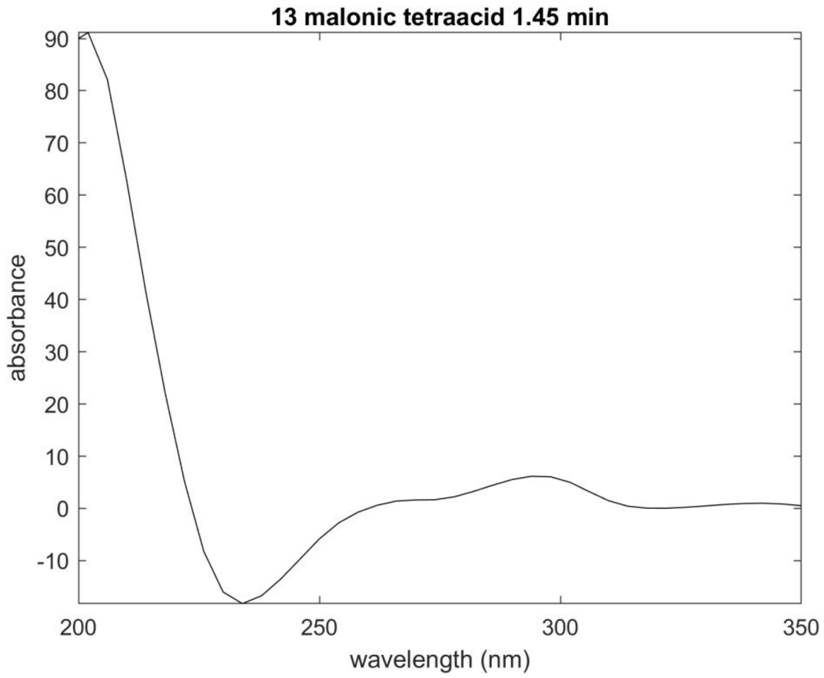

Figure SI52: *UV-visible spectrum for malonic tetraacid* ***13*** *retention time 1.45 minutes.*

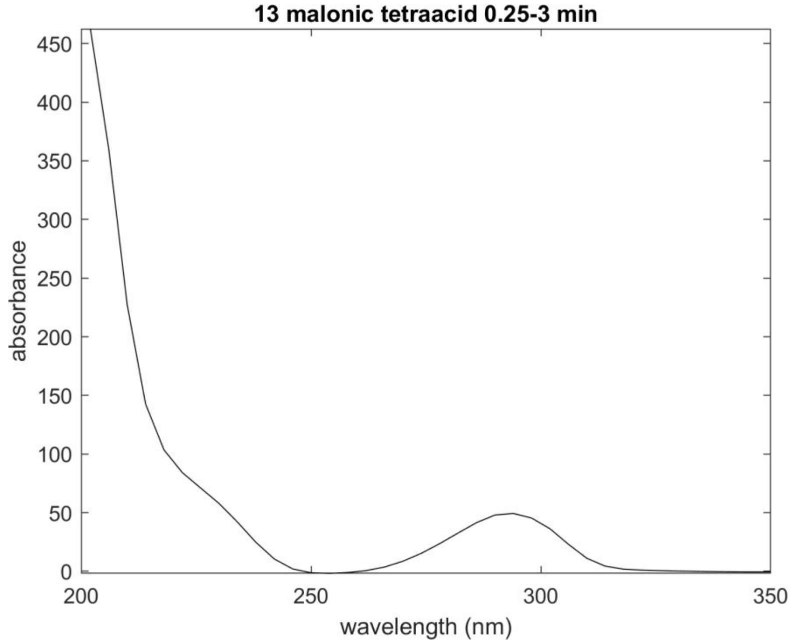

Figure SI53: *UV-visible spectrum for total LC chromatogram of malonic tetraacid* ***13****.*

References

(1) Craig, A. J.; Moodie, L. W.; Hawkes, J. A. Preparation of Simple Bicyclic Carboxylate-Rich Alicyclic Molecules for the Investigation of Dissolved Organic Matter. *Environ. Sci. Technol.* **2024**, *58* (16), 7078–7086.

(2) Kester, D. R.; Duedall, I. W.; Connors, D. N.; Pytkowicz, R. M. Preparation of Artificial Seawater 1. *Limnol Ocean.* **1967**, *12* (1), 176–179.

(3) *https://sunclimate.gsfc.nasa.gov/article/solar-irradiance (accessed 03-02-2025)*.

(4) *CIE 85: Solar Spectral Irradiance*; International Commission on Illumination (CIE), 1989. https://cie.co.at/publications/solar-spectral-irradiance (*accessed 30-07-2025)*.

(5) Breyer, E.; Espada-Hinojosa, S.; Reitbauer, M.; Karunarathna, S. C.; Baltar, F. Physiological Properties of Three Pelagic Fungi Isolated from the Atlantic Ocean. *J. Fungi* **2023**, *9* (4), 439.
